# Integrated Analysis of Physiological, mRNA Sequencing, and miRNA Sequencing Data Reveals a Specific Mechanism for the Response to Continuous Cropping Obstacles in *Pogostemon cablin* Roots

**DOI:** 10.3389/fpls.2022.853110

**Published:** 2022-04-01

**Authors:** Wuping Yan, Shijia Cao, Yougen Wu, Zhouchen Ye, Chan Zhang, Guanglong Yao, Jing Yu, Dongmei Yang, Junfeng Zhang

**Affiliations:** College of Horticulture, Hainan University, Haikou, China

**Keywords:** *Pogostemon cablin* (patchouli), root, continuous cropping obstacles, miRNA, mRNA, response mechanism

## Abstract

*Pogostemon cablin* (patchouli) is a commercially important medicinal and industrial crop grown worldwide for its medicinal and aromatic properties. Patchoulol and pogostone, derived from the essential oil of patchouli, are considered valuable components in the cosmetic and pharmaceutical industries. Due to its high application value in the clinic and industry, the demand for patchouli is constantly growing. Unfortunately, patchouli cultivation has suffered due to severe continuous cropping obstacles, resulting in a significant decline in yield and quality. Moreover, the physiological and transcriptional changes in patchouli in response to continuous cropping obstacles remain unclear. This has greatly restricted the development of the patchouli industry. To explore the mechanism underlying the rapid response of patchouli roots to continuous cropping stress, integrated analysis of the transcriptome and miRNA profiles of patchouli roots under continuous and noncontinuous cropping conditions in different growth periods was conducted using RNA sequencing (RNA-seq) and miRNA-seq and complemented with physiological data. The physiological and biochemical results showed that continuous cropping significantly inhibited root growth, decreased root activity, and increased the activity of antioxidant enzymes (superoxide dismutase, peroxidase, and catalase) and the levels of osmoregulators (malondialdehyde, soluble protein, soluble sugar, and proline). Subsequently, we found 4,238, 3,494, and 7,290 upregulated and 4,176, 3,202, and 8,599 downregulated differentially expressed genes (DEGs) in the three growth periods of continuously cropped patchouli, many of which were associated with primary carbon and nitrogen metabolism, defense responses, secondary metabolite biosynthesis, and transcription factors. Based on miRNA-seq, 927 known miRNAs and 130 novel miRNAs were identified, among which 67 differentially expressed miRNAs (DEMIs) belonging to 24 miRNA families were induced or repressed by continuous cropping. By combining transcriptome and miRNA profiling, we obtained 47 miRNA-target gene pairs, consisting of 18 DEMIs and 43 DEGs, that likely play important roles in the continuous cropping response of patchouli. The information provided in this study will contribute to clarifying the intricate mechanism underlying the patchouli response to continuous cropping obstacles. In addition, the candidate miRNAs and genes can provide a new strategy for breeding continuous cropping-tolerant patchouli.

## Introduction

*Pogostemon cablin* (Blanco) Benth (patchouli), a commercially important medicinal and industrial crop, is a perennial aromatic herbaceous species belonging to the Lamiaceae (Labiatae) family. It is native to South and Southeast Asia and was brought to China as early as during or possibly before the Liang Dynasty for perfume and medicinal purposes (Wu et al., 2007). Dried aerial parts of patchouli (Pogostemonis Herba, also known as “Guanghuoxiang”) have been widely used in traditional Chinese medicine for their ability to stimulate appetite, stop vomiting, eliminate dampness with aromatics, and clear summer heat (China Pharmacopoeia Committee, 2015). In India, the plant is included in the preparations of Ayurvedic treatments such as Rasa, Guna, and Virya (Kumara and Sinniah, 2015). In China, Japan, and Malaysia, it is used to treat colds, headaches, nausea, vomiting, diarrhea, abdominal pain, and insect and snake bites (Chakrapani et al., 2013). Additionally, patchouli oil, extracted from the dried aerial parts of the plant, contains high amounts of patchouli alcohol (patchoulol, PA) and pogostone (dhelwangin, PO) and is an important ingredient in the perfume industry, where it is used as a base to provide long-lasting properties to scents (Zhang J. et al., 2018). Because of its aromatic properties, patchouli oil is also widely used in aromatherapy to mitigate depression and anxiety, calm nerves and improve sexual interest (Swamy and Sinniah, 2016). Patchouli is reported to be one of the 20 essential oil-producing plants that are traded on a regular basis on the global market and has enormous economic potential (Singh et al., 2015). As a result of their clinical and industrial importance, patchouli plants are well known throughout the world. Despite the fact that the species has been widely cultivated in tropical and subtropical Asian countries such as India, Malaysia, China, and the Philippines, the global market demand for patchouli has not been met (Swamy and Sinniah, 2016). According to Lawrence (2009), the annual global patchouli herb production for essential oil is estimated to be approximately 1,200 tons, but the global patchouli demand is 1,600 tons of oil per year (Swamy et al., 2015).

Patchouli is currently grown primarily in southern China, with the primary patchouli-growing districts being Guangdong Province and Hainan Province. According to the different growing regions in China, patchouli cultivars are classified as *P. cablin* Nanxiang (NX, grown in Hainan), *P. cablin* Paixiang (PX, grown in Guangzhou), *P. cablin* Zhanxiang (ZNX, grown in Zhanjiang), or *P. cablin* Zhaoxiang (ZX, grown in Zhaoqing; Wu et al., 2010). Our long-term investigations have revealed that the planting areas of PX and ZX had decreased drastically because of city development, cultivated land compression, and species retrogression. Unfortunately, although NX and ZNX have larger cultivation areas, there are obvious obstacles associated with continuous cropping (Xu et al., 2015; Zhang J. et al., 2018; Zeng et al., 2020). The patchouli root system deteriorates, turns brown, and decays as a result of continuous cropping, and the leaves become weak, yellow, and withered, exposing the plant to serious diseases, resulting in a significant decrease in yield and quality (He et al., 2018). In commercially standardized cultivation, most farmers sidestep the problem by rotating crops or leaving areas uncultivated (Li et al., 2020). Both of these methods can decrease the yield of patchouli. Furthermore, some farmers tend to increase fertilizer inputs and pesticide use to enhance crop yield, leading to an exacerbated soil environment with excessive pesticide residues (Xu et al., 2015).

Continuous cropping refers to crops being farmed in the same field year after year, resulting in lower crop yields, quality deterioration, poor growth, and disease aggravation even under normal cultivation management measures; these problems are also known as soil sickness, replant disease, and consecutive monoculture problems (He et al., 2019; Li et al., 2019). These problems are typically observed in agricultural crops, especially medicinal plants, such as *Panax ginseng* (Tong et al., 2021), *Panax notoginseng* (Tan et al., 2017), *Rehmannia glutinosa* (Li M. et al., 2017; Zhang et al., 2020), *Lepidium meyeni* (Wang et al., 2019), and *Andrographis paniculata* (Li et al., 2020), severely hindering the production, and quality of crops. A number of studies on related continuous cropping obstacles have suggested that allelopathic autotoxicity, aggravation of soil-borne diseases, increase in nematode abundance, deterioration of soil physicochemical properties, and imbalance of soil nutrients are the main causes underlying the continuous cropping obstacles (Zhang et al., 2020). Presently, some scholars have conducted studies on the continuous cropping obstacles of patchouli, but the underlying mechanism remains unclear (Xu et al., 2015; Zhang J. et al., 2018).

The continuous cropping obstacles are a complex form of stress that severely hinders the sustainable development of medicinal plant resources. Previous studies have shown that miRNAs regulate almost all life processes in plants, such as growth and development (Zhang et al., 2017), metabolism (Zhao et al., 2020), hormone signaling (Liu and El-Kassaby, 2017), and the stress response (Burkhardt and Day, 2016; Chen et al., 2020). Plants can induce the expression of certain miRNAs under various stresses, and these miRNAs can act on stress-related target genes, allowing plants to adapt to stress physiologically (Pagano et al., 2021). For instance, overexpression of miR408 in transgenic chickpea significantly increased drought tolerance (Hajyzadeh et al., 2015). In the germination and seedling phases, a rice dh mutant with osa-miR171c overexpression showed a significant reduction in salt tolerance (Yang et al., 2017). The negative-regulatory effects of miR4243-x and novel-m064-5p on the shikimate O-hydroxycinnamoyl transferase (HCT) and sucrose-phosphate synthase (SPS) genes, respectively, were involved in the response of potato to alkali stress (Kang et al., 2021). Rapid advancements in next-generation high-throughput sequencing (HTS) technology and analytic tools have enabled the identification of conserved and new miRNAs in medicinal plants in recent decades (Ye et al., 2020; Gutiérrez-García et al., 2022). China is one of the countries with the most abundant germplasm resources of medicinal plants in the world, but with the intensification of research, the demand for such resources is also increasing (Huang, 2011). The yield of wild medicinal plants is generally low and is easily affected by the external environment (Chen et al., 2016). Therefore, studying miRNAs related to the stress response in medicinal plants is critical. Yang et al. (2011) and Li et al. (2013) compared miRNAs and their mRNA targets in *R. glutinosa* between noncontinuous cropping and continuous cropping conditions and suggested that the miRNAs were likely involved in the continuous cropping obstacles. Zhang H. et al. (2016) identified 31 miRNAs from 14 miRNA families, including one novel miRNA responding to *Salvia miltiorrhiza* during continuous cropping.

Plant miRNAs regulate gene expression at the posttranscriptional level based on the principle of sequence complementarity *via* two major mechanisms: target mRNA cleavage and translation repression (Yu et al., 2017). Elucidation of the miRNA-mRNA regulatory networks present under various biotic and abiotic stresses can initially reveal the role of miRNAs in the gene network regulating plant stress resistance. The use of integrated miRNA-mRNA analysis to study stress response in medicinal plants (Jung et al., 2018; Chen et al., 2020; Yang et al., 2021) has significantly increased our understanding of the mechanisms of stress response in medicinal plants. In this study, we simultaneously profiled mRNA and miRNA expression in the roots at the three growth stages of patchouli under both continuous cropping and noncontinuous cropping by HTS technology and further performed an integrated analysis of mRNA and miRNA expression to identify target genes regulated by specific miRNAs and to construct a gene expression regulatory network for patchouli roots under continuous cropping, aiming to reveal the molecular mechanisms associated with the continuous cropping response. To the best of our knowledge, this is the first report on integrated analysis of mRNA sequencing (RNA-seq) and miRNA sequencing (miRNA-seq) data for patchouli and thus offers deeper insight into the molecular response mechanisms of patchouli roots under continuous cropping. Furthermore, we explored the impact of continuous cropping on some physiological and biochemical traits of patchouli roots. In this study, we used transcriptomics and physio-biochemical characterization to explore the molecular and physio-biochemical response mechanisms of patchouli roots under continuous cropping. These results will help to improve the continuous cropping tolerance of patchouli and provide a theoretical basis for further overcoming continuous cropping-related problems in plants.

## Materials and Methods

### Plant Materials and Continuous Cropping Treatment

The experimental material in this study was *P. cablin* NX, a high-PA-accumulating cultivar that was strongly affected by continuous cropping obstacles (Xu et al., 2015; Zhang J. et al., 2018). The branches of patchouli with robust growth and no plant diseases and insect pests were selected for cutting propagation. After 30 days of cutting, the cutting seedlings with good root growth and uniform plant growth were selected and transplanted to land where patchouli had not been planted in the past 10 years (1st-year plant, FP) and to land where the same crop had been grown the previous year (2nd-year plant, SP); the planting cycle was 9 months. All other management measures were identical for both treatments, and the two planting areas were next to each other. According to variations in plant size, color, and biomass, patchouli growth and development can be split into four main phases: slow growth period (0–120 days, S1), fast-growth period (120–180 days, S2), ripe period (180–210 days, S3), and fully ripe period (210–270 days, S4; Chen et al., 2014). In the fast-growth period, ripe period, and fully ripe period of patchouli growth, fresh root samples were obtained from five independent plants and combined as one biological repetition. In each phase, there were three biological duplicates for each treatment, yielding a total of 18 root samples (FP_S2, FP_S3, FP_S4, SP_S2, SP_S3, and SP_S4, each with three biological replicates). After promptly washing all the root samples with sterile water, they were frozen in liquid nitrogen and stored in a −80°C freezer until needed. We used identical cultivation and sample collection procedures for physiological, biochemical, and sequencing analyses.

### Determination of Physiological and Biochemical Traits

The root samples of the FP and SP patchouli in the S2, S3, and S4 periods were used to measure root activity, antioxidant metabolism, and osmoregulatory substance levels. The malondialdehyde (MDA) content, soluble sugar (SS) content, soluble protein (SPr) content, proline (Pro) content, superoxide dismutase (SOD) activity, peroxidase (POD) activity, and catalase (CAT) activity were investigated with assay kits (MDA-2-Y, KT-2-Y, KMSP-2-W, PRO-2-Y, SOD-2-W, POD-2-Y, and CAT-2-W) from Suzhou Keming Biotechnology Co. Ltd. (Suzhou, China) according to the manufacturer’s protocols. The triphenyltetrazolium chloride (TTC) reduction method was used to determine root activity (Chen et al., 2018).

### RNA Isolation, Library Construction, and Sequencing

The total RNA of patchouli roots was extracted following the manufacturer’s procedure using the mirVana miRNA Isolation Kit (Ambion, AM1561). Next, following the manufacturer’s recommendations, 18 mRNA libraries were created using the TruSeq Stranded mRNA LTSample Prep Kit (Illumina, San Diego, CA, United States). In addition, 1 mg of total RNA was pipetted from each sample to construct the small-RNA (sRNA) library using the TruSeq Small-RNA Sample Prep Kit (Cat. No. RS-200-0012; Illumina, United States) according to the manufacturer’s instructions. Finally, transcriptome and sRNA sequencing were carried out on an Illumina HiSeq X Ten platform by OE Biotech Co., Ltd. (Shanghai, China).

### Expression Analysis of mRNAs

First, we used Trimmomatic to cope with raw data (raw reads) in fastq format (Bolger et al., 2014). Subsequently, clean reads were obtained by removing the adapter sequence, reads containing Poly-N sequence, and low-quality reads. HISAT2 (Kim et al., 2015) was used to align the clean reads to the patchoul reference genome (PRJNA471952, He et al., 2018). Htseq count (Anders et al., 2015) was used to estimate the read counts per gene, and the fragments per kilobase per million mapped reads (FPKM, Trapnell et al., 2010) value per gene was calculated using cufflinks. The DESeq R package (Anders and Huber, 2012) was used to perform differential expression analysis between groups using read counts. The thresholds for significant differential expression were set at a |log2 Fold Change| ≥1 and value of *p* ≤ 0.05. To demonstrate the expression patterns of genes in different groups and samples, hierarchical cluster analysis of differentially expressed genes (DEGs) was carried out. To further assess the biological functions of these DEGs, Gene Ontology (GO) and Kyoto Encyclopedia of Genes and Genomes (KEGG) enrichment analysis on these DEGs were carried out using the GOseq R package (Young et al., 2010) and KOBAS 2.0.12 software (Mao et al., 2005), respectively.

### Expression Analysis of miRNAs

Base calling was used to convert the basic reads to sequence data (also known as raw data/reads). Low-quality reads, reads containing poly (A), and contaminated reads were deleted, and reads less than 15 nt and greater than 41 nt were filtered out from the original data to obtain clean reads. The length distribution of clean reads was statistically analyzed to initially assess the distribution of sRNAs in the samples. Clean reads were aligned to the reference genome of patchouli (PRJNA471952, He et al., 2018), and the percentage of reads mapped to the genome was calculated. Noncoding RNAs were annotated as rRNAs, tRNAs, Cis-regs, small nuclear RNAs (snRNAs), other Rfam RNAs, repeats, and so on. These RNAs were aligned and then searched against the Rfam v.10.1^1^ (Sam et al., 2003) and GenBank databases^2^ using BLAST (Altschul et al., 1990). Alignment was performed against the miRBase V22 database^3^ to identify known miRNAs (Griffiths-Jones et al., 2008) and analyze known miRNA expression patterns in different samples. Then, mirdeep2 (Friedländer et al., 2012) was used to analyze the unannotated reads for prediction of novel miRNAs. Based on the hairpin secondary structure of miRNA precursors, we combined the miRBase database results to identify the corresponding miRNA star sequences and mature miRNA sequences. The transcripts per kilobase per million mapped reads (TPM) value was used to compute and standardize the expression levels of all identified miRNAs, as described elsewhere (Zhang X. et al., 2018; Zhu et al., 2020). Differential expression analysis of the 18 samples was performed using the DEGseq R program based on read counts. miRNAs that had a value of *p*< 0.05 and a |log2(fold change)| more than one were considered significantly differentially expressed. In addition, Targetfinder (Fahlgren and Carrington, 2010) was used to predict miRNA targets in plants. Based on the hypergeometric distribution, R was used to perform GO enrichment and KEGG pathway enrichment analyses of differentially expressed miRNA (DEMI) target genes. The miRNAs and their target genes were statistically analyzed, and Cytoscape was used to create a miRNA-mRNA network.

### Validation of DEG and DEMI Expression by qRT-PCR

To verify the authenticity of the sequencing data, 18 DEGs and seven DEMIs were randomly selected for quantitative real-time PCR (qRT-PCR) analysis. Additionally, six pairs of miRNA-mRNA were selected for qRT-PCR analysis. The qRT-PCR primers for DEG and DEMI analysis were synthesized using Primer Premier 5.0 (Premier Biosoft International, Palo Alto, CA, United States) and are listed in Supplementary Table 1. Total RNA was extracted and purified using the previously described methods. For DEG quantification, the total RNA served as a template to synthesize cDNA using MonScript™ RTIII All-in-One Mix with dsDNase (Monad Biotech Co., Ltd., Shanghai, China). Then, RT-PCR was performed with MonAmp™ ChemoHS qPCR Mix (Monad Biotech Co., Ltd., Shanghai, China). In brief, qRT-PCR was performed in a total volume of 20 μl, containing 10 μl of MonAmp™ ChemoHS qPCR Mix, 0.4 μl each of the forward and reverse primers, 8.7 μl of sterile double-distilled H_2_O (sddH_2_O), and 0.5 μl of cDNA. The reference 18S ribosomal RNA (18S rRNA) gene was used as an internal control (Yan et al., 2021a). For DEMI quantification, miRNA molecules were polyadenylated and reverse transcribed to cDNA using the miRcute Plus miRNA First-Strand cDNA Kit (TIANGEN, Beijing, China). qRT-PCR analysis was conducted using the miRcute Plus miRNA qPCR Kit (SYBR Green, TIANGEN, Beijing, China) with reverse adaptor primers (10 μM). U6 small nuclear RNA (U6 snRNA) was used as a reference gene. qRT-PCR for the selected mRNAs or miRNAs was performed on a Roche LightCycler®96 system (Roche, Switzerland), and each experiment included three technical replicates to ensure reproducibility. The relative levels of DEGs or DEMIs were computed using the 2^−ΔΔCt^ method (Livak and Schmittgen, 2001).

### Statistical Analysis

All physiological and biochemical data are expressed as the mean ± SD of three independent biological replicates. Data processing and analysis were performed using SPSS 20 (International Business Machines Inc., United States) and Microsoft Excel 2010 (Microsoft Corp., United States).

## Results

### Phenotypic and Physiological Responses to Continuous Cropping Obstacles

**Figure 1:**
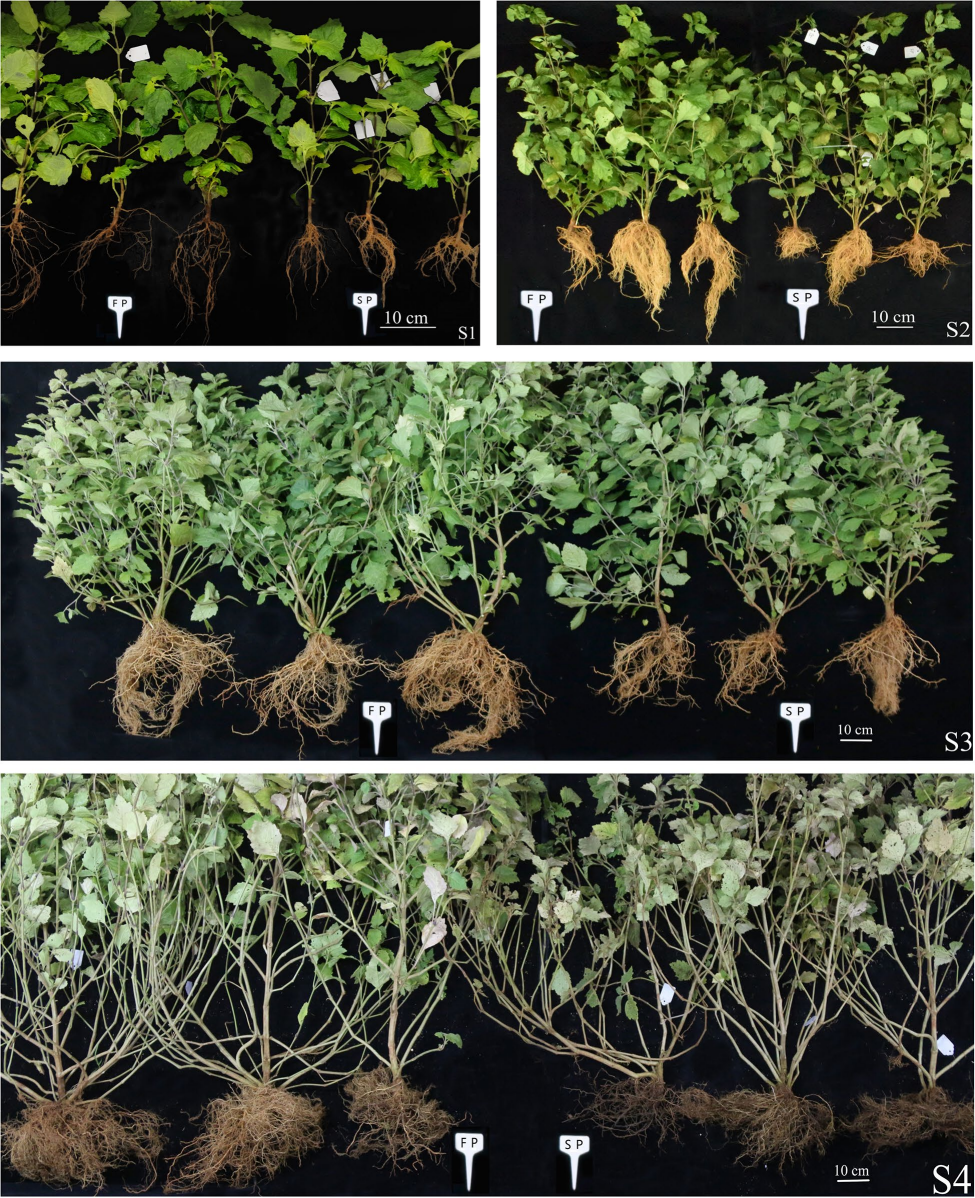
Comparison of the phenotypes of SP and FP patchouli at four growth stage. S1: slow growth period (60 days), S2: fast-growth period (150 days), S3: ripe period (195 days), and S4: fully ripe period (255 days).

The growth and development of patchouli were severely inhibited under continuous cropping stress, as evidenced by the lower below- and aboveground biomass in continuous cropping patchouli compared to plants grown normally (Figure 1). The morphology of patchouli roots changed with increasing continuous cropping duration. In the S1 period, no significant difference was found in the morphology and physiology of the below- and aboveground parts of the SP and FP patchouli plants. However, the phenotype of the below- and aboveground parts of SP patchouli exhibited inconsistent changes in the S2 period. During this period, the root growth of SP patchouli began to be inhibited, but the growth of the aboveground part was not inhibited. In accordance with the phenotypic changes of patchouli roots, the root activity decreased, and the antioxidant enzyme (POD, SOD, and CAT) activity and the levels of osmoregulatory substances (MDA, SP, SS, and Pro) increased during this period in SP patchouli (Table 1). After the S2 period, the morphology and physiology of the below- and aboveground parts of SP and FP patchouli began to change significantly. The root growth of SP patchouli was significantly inhibited, the roots became increasingly shorter, and the color became darker. In particular, in the S4 period, a few roots began to brown and rot. Moreover, the root activity decreased significantly, and the activity of antioxidant enzymes and the levels of osmoregulatory substances increased significantly. These results showed that the period during which continuous cropping obstacles were most likely to cause damage was the fast-growth period. Therefore, we next performed RNA-seq and miRNA-seq on the root samples of SP and FP patchouli plants in the S2, S3, and S4 periods to explore the mechanism underlying the rapid response of patchouli roots to continuous cropping stress.

**Table 1:** Physiological responses of patchouli roots under continuous cropping stress.

Sample	POD (U/g, FW)	SOD (U/g, FW)	CAT (μmol/min/g, FW)	MDA (nmol/g, FW)	Pro (μg/g, FW)	SS (mg/g, FW)	SPr (mg/g, FW)	Root activity (μg/g/h, FW)
FP_S1	10463.94 ± 617.63^D,a^	218.48 ± 20.55^A,a^	3.25 ± 0.25^C,a^	12.83 ± 1.16^B,a^	14.15 ± 0.25^C,a^	6.29 ± 0.16^D,a^	0.6 ± 0.07^B,a^	85.41 ± 12.2^A,a^
SP_S1	12806.8 ± 749.15^D,b^	377.24 ± 14.38^C,b^	3.75 ± 0.42^B,a^	12.83 ± 0.57^A,B,a^	14.54 ± 0.58^B,a^	6.92 ± 0.09^C,b^	0.64 ± 0.12^A,a^	88.97 ± 6.96^A,a^
FP_S2	3520.34 ± 144.29^A,a^	270.73 ± 20.82^B,a^	2.69 ± 0.31^B,a^	9.75 ± 0.83^A,a^	10.36 ± 0.44^B,a^	2.3 ± 0.26^A,a^	0.45 ± 0.05^A,a^	99.15 ± 4.91^A,b^
SP_S2	4322.68 ± 87.94^A,b^	271.84 ± 14.35^A,a^	2.76 ± 0.14^A,a^	11.18 ± 1.55^A,a^	12.27 ± 0.26^A,b^	4.63 ± 0.14^A,b^	0.67 ± 0.02^A,b^	92.87 ± 2.02^A,a^
FP_S3	5670.5 ± 205.93^B,a^	195.23 ± 14.36^A,a^	1.75 ± 0.02^A,a^	11.42 ± 0.95^B,a^	9.14 ± 0.63^A,a^	3.91 ± 0.17^C,a^	0.52 ± 0.03^A,B,a^	150.93 ± 9.51^B,c^
SP_S3	6709.75 ± 190.82^B,b^	274.32 ± 21.11^A,b^	2.7 ± 0.25^A,b^	12.27 ± 1.71^A,a^	11.64 ± 0.19^A,b^	4.73 ± 0.15^A,b^	0.58 ± 0.01^A,a^	102.42 ± 11.65^A,b^
FP_S4	9186.25 ± 311.8^C,a^	205.29 ± 12.92^A,a^	3.39 ± 0.15^C,a^	12.31 ± 0.33^B,a^	10.31 ± 0.39^B,a^	3.43 ± 0.24^B,a^	0.59 ± 0.03^B,a^	225.79 ± 16.51^B,d^
SP_S4	10449.73 ± 276.53^C,b^	320.88 ± 16.56^B,b^	4.89 ± 0.36^C,b^	15.24 ± 1.06^B,b^	16.26 ± 1.26^C,b^	5.96 ± 0.09^B,b^	0.68 ± 0.02^A,b^	152.62 ± 16.02^A,c^

All data are presented as means ± SE from three independent experimental replicates. Different capital letters indicate significantly different at *p* < 0.05 between different periods of the same treatment, different lowercase letters indicate significantly different at *p* < 0.05 between different treatments at the same period. POD, peroxidase; SOD, superoxide dismutase; CAT, catalase; MDA, malondialdehyde; Pro, proline; SS, soluble sugar; and SPr, soluble protein.

### Differential Expression in the Transcriptome in Response to Continuous Cropping

To explore the influence of continuous cropping on gene expression in different growth periods of patchouli, we performed RNA-seq analysis during three growth periods (S2, S3, and S4) of FP and SP patchouli plants. A total of 18 cDNA libraries were constructed and sequenced, and the raw and clean reads are presented in Supplementary Table 2. According to our sequencing results, the raw data size of the 18 samples ranged from 7.09 GBases to 7.76 GBases, with a total of 133.14 GBases. Approximately 121.95 GBase clean bases (ranging from 6.42 GBases to 7.17 GBases in each sample) were obtained after removing the adaptor sequences, low-quality reads, and reads containing more than 5% N bases. The average Q30 base and GC percentages were 94.30% and 45.55%, respectively. The transcriptome characteristics of 18 samples were compared intuitively using a correlation heat map and cluster dendrogram (Supplementary Figure 1). As shown, the SP and FP samples in the same growth period were clustered together, indicating that there was no significant difference in gene expression between these samples. In addition, the Pearson’s correlation coefficient (R value) of three biological replicates for each sample was greater than 90%, indicating high consistency between replicates.

**Figure 2:**
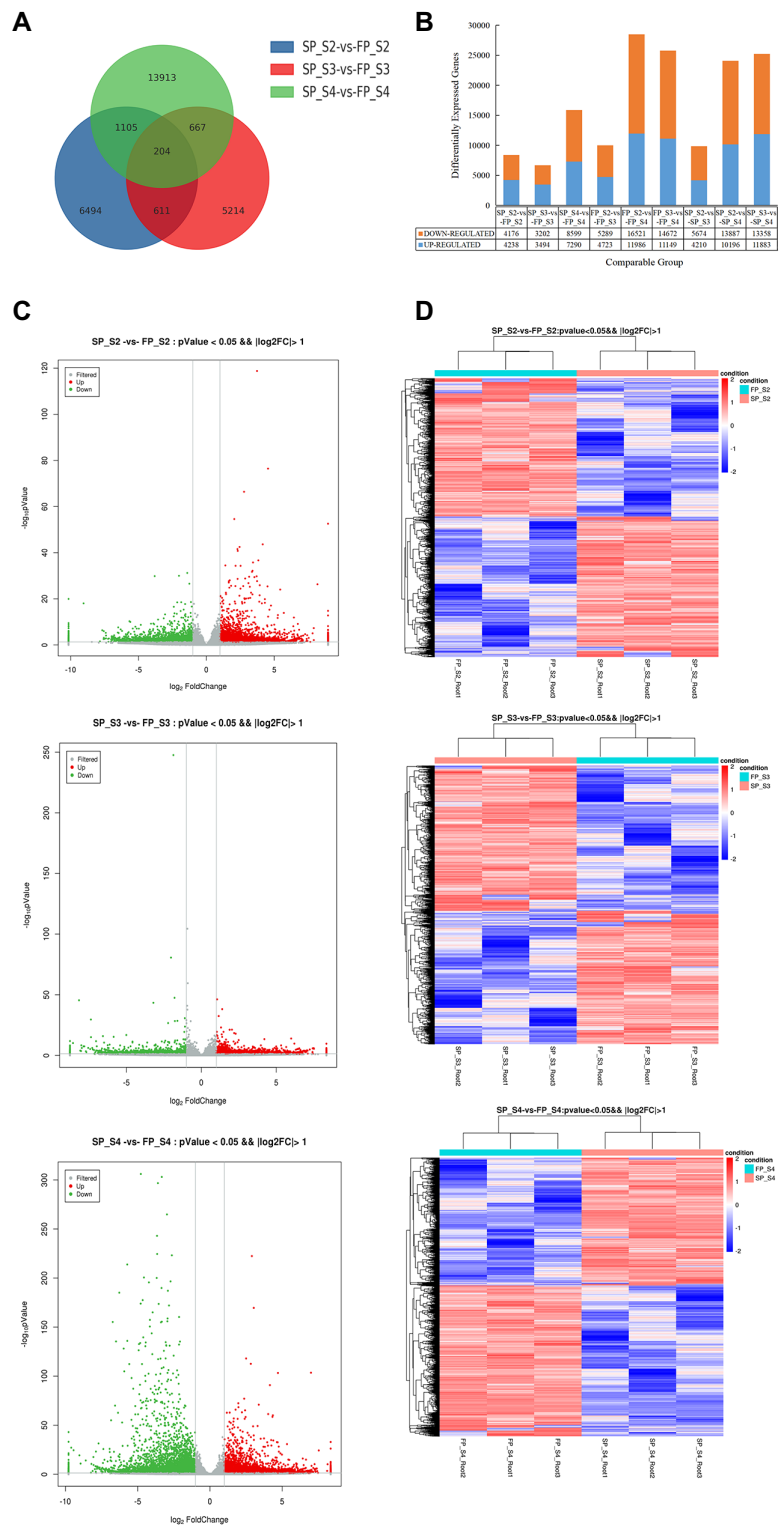
Expression profile of differentially expressed genes (DEGs) at three periods of patchouli roots under continuous cropping stress. **(A)** Numbers of DEGs. **(B)** Venn diagram of the number of DEGs at each periods under continuous cropping stress. **(C)** Volcano plot. Each point in the volcano plot represents a gene, the green points represent downregulated genes, the red points represent upregulated genes, and the gray points represent unchanged genes. **(D)** Hierarchical clustering analysis of all DEGs. Different columns in the figure represent different samples, and different rows represent different genes. Colors from blue to red indicate gene expression from low to high, respectively.

The expression level in each sample was assessed based on the overall discrete expression level (Supplementary Figure 2). To reveal the transcriptional responses of patchouli roots to continuous cropping stress, we compared the transcriptional profiles of FP and SP in three different growth periods of patchouli development and identified DEGs in each period. The number of DEGs in each comparison group is shown in Figure 2B. Compared with FP, 8414, 6,696, and 15,889 DEGs were detected under continuous cropping in S2, S3, and S4, respectively. The highest number of DEGs was observed in the S4 period, 2.37-fold higher than that in the S3 period. There were 4,238, 3,494, and 7,290 upregulated and 4,176, 3,202, and 8,599 downregulated DEGs in S2, S3 and S4, respectively (Figures 2C,D). In these three periods, the downregulated DEGs accounted for approximately 51.54% of the total, indicating that continuous cropping inhibited the expression of a greater number of genes. The number of DEGs showing overlaps and specific responses under continuous cropping stress in different growth periods is visualized in Figure 2A. The Venn plot shows that 204 DEGs overlapped among the three comparisons. Eighteen and eight genes were continuously upregulated and downregulated, respectively, in the three periods. The overlap between SP_S2 vs. FP_S2 and SP_S4 vs. FP_S4 was the largest (1,105 DEGs). The overlap between SP_S2 vs. FP_S2 and SP_S3 vs. FP_S3 and that between SP_S3 vs. FP_S3 and SP_S4 vs. FP_S4 had 611 and 667 DEGs, respectively. These results indicated that each stage had unique and independent defense mechanisms to respond to continuous cropping stress.

### Enrichment Analysis of the DEGs

To analyze the functions of these genes in the response to continuous cropping in different growth periods of patchouli, we performed GO enrichment analysis of the DEGs (5,636 in S2, 4,363 in S3, and 10,829 in S4) identified in this study. Most of the DEGs were mainly enriched in the cell, cell part, and organelle terms in cellular component and in the cellular process, metabolic process, response to stimulus in biological process, binding, catalytic activity, and transporter activity terms in molecular function (Supplementary Figure 3). Moreover, 824, 820, and 952 GO terms were significantly enriched (value of *p* < 0.05) in SP_S2 vs. FP_S2, SP_S3 vs. FP_S3, and SP_S4 vs. FP_S4, respectively. There were 77 common significantly enriched GO terms in the above three pairwise comparisons, including 35 biological process terms, 4 cellular component terms, and 38 molecular function terms. Among them, the top 5 GO terms with the highest number of DEGs were “extracellular region” (GO:0005576), “aspartic-type endopeptidase activity” (GO:0004190), “cell wall” (GO:0005618), “apoplast” (GO:0048046), and “carbohydrate metabolic process” (GO:0005975). The 30 most significant terms in the GO enrichment analysis of DEGs are shown in Supplementary Figure 4. The top 3 significantly enriched GO terms of upregulated DEGs in S2 were “(−)-exo alpha bergamotene biosynthetic process” (GO:1901940), “farnesyl diphosphate catabolic process” (GO:0045339), and “sesquiterpene synthase activity” (GO:0010334). The top 3 significantly enriched GO terms of downregulated DEGs in S2 were “response to hydrogen peroxide” (GO:0042542), “protein complex oligomerization” (GO:0051259), and “water channel activity” (GO:0015250). “FAD binding” (GO:0071949), “(−)-exo-alpha-bergamotene biosynthetic process,” “farnesyl diphosphate catabolic process,” and “plant-type secondary cell wall biogenesis” (GO:0009834), “cellulose biosynthetic process” (GO:0030244), and “cellulose synthase activity” (GO:0016759) were the top 3 significantly enriched GO terms of upregulated DEGs in S3 and S4, respectively. “Oligopeptide transport” (GO:0006857), “inositol-3-phosphate synthase activity” (GO:0004512), “all-trans-beta-apo-10′-carotenal cleavage oxygenase activity” (GO:0102251), “RNA secondary structure unwinding” (GO:0010501), “chloroplast stromal thylakoid” (GO:0009533), and “methionyl glutamyl tRNA synthetase complex” (GO:0017102) were the top 3 significantly enriched GO terms of downregulated DEGs in S3 and S4, respectively.

To further assess the biological functions of these DEGs in patchouli under continuous cropping stress, we performed KEGG pathway analysis of the DEGs in SP and FP in the same growth period. A total of 127, 123, and 128 pathways were categorized by pairwise comparison of the enrichment analysis results for KEGG pathways in SP_S2 vs. FP_S2, SP_S3 vs. FP_S3, and SP_S4 vs. FP_S4, respectively. Among them, 25, 15, and 45 pathways were significantly enriched (value of *p* < 0.05) in SP_S2 vs. FP_S2, SP_S3 vs. FP_S3, and SP_S4 vs. FP_S4, respectively. The top 20 enriched KEGG pathways for each comparison are shown in Supplementary Figure 5. In the S2 period, the most enriched pathway for upregulated DEGs was plant-pathogen interaction (ko04626), in which 57 upregulated DEGs were enriched in it. In the plant-pathogen interaction pathway, genes encoding calmodulin and related proteins, Ca^2+^/calmodulin-dependent protein kinase, calcium-dependent protein kinase, and calmodulin-like protein were significantly upregulated in continuous cropping patchouli, followed by protein processing in endoplasmic reticulum (ko04141) pathway, with 56 upregulated differential genes enriched. Among them, the genes encoding E3 ubiquitin-protein ligase and dolichyl-diphosphooligosaccharide-protein glycosyltransferase were significantly upregulated in continuous cropping patchouli. Many upregulated DEGs were enriched in plant hormone signal transduction (ko04075) and glycolysis/gluconeogenesis (ko00010) pathways, such as genes encoding ethylene receptor, serine/threonine-protein kinase, abscisic acid receptor, auxin response factor, and alcohol dehydrogenase. The most enriched pathway for downregulated DEGs was protein processing in endoplasmic reticulum (ko04141), such as 46 genes encoding heat shock proteins and 4 genes encoding molecular chaperones. Additionally, many downregulated DEGs were enriched in galactose metabolism (ko00052), Spliceosome (ko03040), isoquinoline alkaloid biosynthesis (ko00950), and inositol phosphate metabolism (ko00562), such as encoding galactinol-sucrose galactosyltransferase, stachyose synthase, splicing factor, serine/arginine-rich splicing factor, polyphenol oxidase, and phosphatidylinositol 4-phosphate 5-kinase. During the S3 period, many upregulated DEGs were mainly enriched in the mRNA surveillance pathway (ko03015), such as genes encoding cleavage stimulating factor and serine/threonine-protein phosphatase. Additionally, many upregulated DEGs were enriched in glycerolipid metabolism (ko00561), alanine, aspartate and glutamate metabolism (ko00250), glutathione metabolism (ko00480), and spliceosome (ko03040), such as genes encoding alpha-galactosidase, diacylglycerol kinase, asparagine synthase, glutathione S-transferase, ATP-dependent RNA helicase, and splicing factor. The downregulated DEGs were mainly enriched in starch and sucrose metabolism (ko00500) and inositol phosphate metabolism (ko00562) pathways, such as encoding glucose-1-phosphate adenylyltransferase, isoamylase, sucrose-phosphate synthase, myo-inositol-1-phosphate synthase, phosphatidylinositol—3-phosphatase, and phosphoinositide phosphatase. In the S4 period, the significantly upregulated DEGs were mainly enriched in protein processing in endoplasmic reticulum (ko04141), which contained many genes such as those encoding heat shock cognate protein, luminal-binding protein, oligosaccharyltransferase, and calreticulin, followed by plant-pathogen interaction (ko04626) and spliceosome (ko03040), which contained genes such as those encoding 3-ketoacyl-CoA synthase, calcium-binding protein, serine/threonine-protein kinase, very-long-chain 3-oxoacyl-CoA synthase, DEAD-box ATP-dependent RNA helicase, heterogeneous nuclear ribonucleoprotein, mediator of RNA polymerase II transcription subunit 35-like protein, serine/arginine-rich splicing factor, and other genes. Additionally, many upregulated DEGs were significantly enriched in plant hormone signal transduction (ko04075), MAPK signaling pathway—plant (ko04016), cysteine and methionine metabolism (ko00270), and phenylpropanoid biosynthesis (ko00940), which contained genes such as those encoding auxin response factor, transcription Factor TGA, mitogen-activated protein kinase, leucine-rich receptor-like protein kinase, 5-methyltetrahydropteroyltriglutamate-homocysteine methyltransferase, S-adenosylmethionine synthase, peroxidase, and phenylalanine ammonia-lyase. The most significant pathway enriched for downregulated DEGs was glycolysis/gluconeogenesis (ko00010), which contained genes such as those encoding pyruvate decarboxylase, pyruvate kinase, thiamine pyrophosphate-requiring enzyme, and fructose-biphosphate aldolase. Many downregulated DEGs were also enriched in plant-pathogen interaction (ko04626) and starch and sucrose metabolism (ko00500), which contained genes such as encoding respiratory burst oxidase homolog protein, calcium-dependent protein kinase, apoptotic ATPase, glucose-1-phosphate adenylyltransferase, sucrose-phosphate synthase, sucrose synthase, and trehalose-6-phosphate synthase. Additionally, many genes, including those encoding serine/threonine-protein kinase, shaggy-related protein kinase, receptor-like protein kinase, and mitogen-activated protein kinase, were significantly activated in continuous cropping patchouli. By contrast, genes encoding ABC transporter, beta-glucosidase, and tripeptidyl-peptidase II were significantly inhibited in the roots of continuous cropping patchouli.

### Differentially Expressed TFs

From 28,208 DEGs, a total of 1,417 DEGs encoding transcription factor (TF) family proteins, including ARF, AP, ATHB, bHLH, MYB, ERF, bZIP, HSF, SCL, LBD, SPL, TCP, NAC, and WRKY TFs, were identified in patchouli roots under continuous cropping stress (Table 2). These TFs were classified into 59 different TF families. The top five TF families containing the greatest number of TFs were the AP2/ERF-ERF, MYB, bHLH, bZIP, and WRKY families, including 143, 102, 90, 84, and 83 TFs, respectively. Most of the DEGs encoding AP2/ERF-ERF and bHLH TFs were upregulated, while most of the DEGs encoding bZIP, MYB-related, HSF, GARP-G2-like, and HB-HD-ZIP TFs were downregulated.

**Table 2:** Transcription factor families that were greatly respond to continuous cropping.

TF families	SP_S2 vs. FP_S2		SP_S3 vs. FP_S3		SP_S4 vs. FP_S4	
	Up	Down	Up	Down	Up	Down
AP2/ERF-ERF	20	11	7	5	63	37
MYB	18	13	11	6	23	31
bHLH	11	16	10	4	31	18
bZIP	3	10	7	10	16	38
WRKY	18	3	3	6	17	36
MYB-related	7	21	10	8	10	19
NAC	11	5	3	2	13	35
Trihelix	12	1	5	7	8	28
GRAS	4	5	2	1	22	15
HSF	4	12	5	2	6	18
C3H	8	8	2	3	14	12
GARP-G2-like	5	7	2	8	10	14
C2H2	5	7	4	2	18	10
HB-HD-ZIP	2	12	2	3	8	15
B3	9	6	5	5	3	14
B3-ARF	2	7	5	4	17	2
LOB	7	2	2	2	10	12
C2C2-Dof	0	11	1	1	12	10
SBP	3	6	5	3	5	11
FAR1	3	10	6	2	3	6
Tify	3	3	5	2	7	6
C2C2-GATA	3	1	1	2	8	9
TCP	2	2	0	2	10	8
MADS-MIKC	3	2	4	3	1	7
HB-other	6	5	1	4	2	2
AP2/ERF-AP2	0	2	2	1	9	4
TUB	2	2	1	1	1	8
C2C2-CO-like	0	4	0	1	5	3
AP2/ERF-RAV	3	3	2	1	0	3
BES1	2	3	2	1	2	1
Others	20	21	14	16	37	38
Total	196	221	129	118	391	470

### Construction and Sequencing of sRNA Libraries

Eighteen sRNA libraries from the three growth periods of FP and SP patchouli roots were constructed. Sequencing results (Supplementary Table 3) showed that 22.41–39.09 million raw reads, with a total of 615.1 million raw reads, were obtained for each of the 18 libraries. After deleting low-quality reads, reads containing poly (A), contaminated reads, and reads less than 15 nt and greater than 41 nt in size, 519.63 million (ranging from 19.57 to 32.79 million in each library) clean reads were obtained from the 18 libraries. The length distribution of the sRNAs showed that the most abundant class of sRNAs was 21-nt sRNAs, followed by 24-nt sRNAs (Supplementary Figure 6A). These sRNAs were categorized and annotated to miRNAs, tRNAs, rRNAs, Cis-regs, snRNAs, repeat regions, and transcript sequences (Supplementary Figure 6B). The annotation results showed that 0.47%–0.83% of the sRNAs were annotated to the previously identified miRNAs, while 85.91%–91.16% of sRNAs were not annotated. The large number of unannotated sRNAs indicated that additional miRNAs remained to be identified in patchouli.

### Identification of Known and Novel miRNAs

We identified 1,057 miRNAs in the three growth periods of FP and SP patchouli roots, including 927 known miRNAs and 130 new miRNAs (Supplementary Table 4). Among them, 870 miRNAs were identified in the three growth periods of FP patchouli roots, including 743 known miRNAs and 127 new miRNAs, and 858 miRNAs were identified in the three growth periods of SP patchouli roots, including 739 known miRNAs and 119 new miRNAs. There were 671 miRNAs shared by FP and SP (Supplementary Figure 6C). One hundred and ninety-nine miRNAs were unique to FP, and 187 miRNAs were unique to SP. The length distribution of 1,057 miRNAs ranged from 18 to 26 nt, and the 21-nt miRNAs were the most abundant, followed by the 22-nt and 20-nt miRNAs (Supplementary Figure 6D). Most of the identified patchouli miRNAs showed significant similarities with several known miRNAs in other plant species. For instance, 83, 70, and 63 of the miRNAs identified were significantly similar to known miRNAs in *Arabidopsis lyrata*, *Glycine max*, and *Oryza sativa*, respectively (Supplementary Figure 7A). Based on sequence homology, 747 (70.67%) of the 1,057 identified miRNAs were identified to belong to 112 miRNA families (Supplementary Table 5). The top 10 miRNA families with the greatest number of miRNA members were MIR159, MIR166, MIR156, MIR171, MIR172, MIR396, MIR167, MIR164, MIR395, and MIR160 (Supplementary Figure 7B). Among them, MIR159, MIR166, MIR156, and MIR171 contained more than 50 miRNAs each. Among these miRNAs identified in patchouli, 28 miRNA families (including MIR162, MIR163, MIR1862, and MIR1023) and three novel miRNAs were expressed in only SP patchouli, and 27 miRNA families (including MIR2118, MIR7486, MIR1027, and MIR1510) were expressed in only FP patchouli. There was a significant difference between miRNA expression levels in the sRNA libraries of SP and FP, indicating the need to explore the relationship between patchouli miRNAs and continuous cropping obstacles.

### Identification of Continuous Cropping-Responsive miRNAs in Patchouli

**Figure 3:**
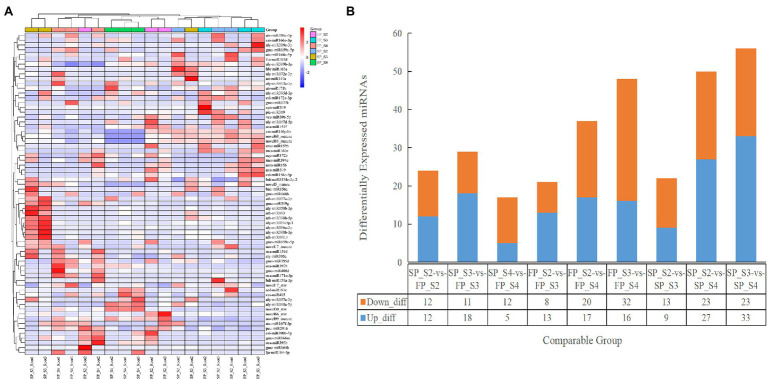
The expression profile of differentially expressed miRNAs (DEMIs) at different growth periods of patchouli roots under continuous cropping stress. **(A)** Hierarchical clustering analysis of DEMIs. **(B)** The number and characteristics of DEMIs among different groups.

The TPM density distribution of novel and conserved miRNAs in the 18 libraries is shown in Supplementary Figure 8. To understand the miRNA regulatory mechanism under continuous cropping obstacles, the DEMIs were identified by the DESeq R package. Based on the thresholds of value of *p* < 0.05 and |log2(fold change)| >1, we identified 24 (12 downregulated and 12 upregulated), 29 (11 downregulated and 18 upregulated), and 17 (12 downregulated and 5 upregulated) DEMIs in SP_S2 vs. FP_S2, SP_S3 vs. FP_S3, and SP_S4 vs. FP_S4, respectively (Figure 3; Supplementary Table 6). A total of 24 miRNA families were identified in these DEMIs. The top 10 DEMIs with the highest expression levels belonged to 6 families: MIR159, MIR168, MIR166, MIR164, MIR403, and MIR167. The most highly upregulated and downregulated miRNAs were aly-miR166a-3p and lja-miR166-3p, respectively, and both were members of the MIR166 family. In addition, two families (MIR159 and MIR167) were found to be differentially expressed in both comparison groups.

### Identification and Analysis of miRNA-Target Genes

miRNAs cannot directly encode proteins and can participate in biological functions only by regulating the expression of target genes. To understand the potential biological functions of the identified DEMIs, we predicted the target genes of miRNAs. Among the 67 DEMIs, 26 DEMIs had 1,318 predicted target genes, and each DEMI regulated 53.12 target genes on average (Supplementary Table 7). A total of 714, 922, and 108 genes were targeted by nine (two novel and seven known), 12 (one novel and 11 known), and five (two novel and three known) DEMIs, respectively, in the three comparison groups. Two DEMIs (novel68_mature and peu-miR2916) had only one predicted target gene, and the remaining DEMIs had multiple predicted target genes. A homology search of these target genes showed that many of them were homologous to genes encoding stress-related TFs, including SPB-like proteins (SPLs), APETALA2 (AP2), MYB domain proteins (MYBs), teosinte branched1/cincinnata/proliferating cell factors (TCPs), auxin response factor (ARF) family proteins, and homeodomain-leucine zipper proteins (HD-ZIPs). In addition, some target genes encoded important enzymes or functional proteins playing a role in a variety of metabolic pathways, such as argonautes (AGOs), ubiquitin-protein ligases (UBCs), laccases (LACs), diacylglycerol kinases (DGKs), lectin receptor kinases (LecRKs), peroxisome biogenesis factor (PEX1), and copper-transporting ATPases (Cu-ATPases).

To determine the potential biological functions of the predicted target genes during the continuous cropping process, GO enrichment analysis was performed. There were 149, 172, and 36 significantly enriched GO terms in SP_S2 vs. FP_S2, SP_S3 vs. FP_S3, and SP_S4 vs. FP_S4, respectively. In the S2 stage, the most significantly enriched GO terms were “lipid binding” (GO:0008289), “lignin catabolic process” (GO:0046274), and “hydroquinone: oxygen oxidoreductase activity” (GO:0052716). In the S3 stage, the most significantly enriched GO terms were “positive regulation of development, heterochronic” (GO:0045962), “positive regulation of nucleic acid-templated transcription” (GO:1903508), and “AP-1 adaptor complex” (GO:0030121). In the S4 stage, the most significantly enriched GO terms were “copper-exporting ATPase activity” (GO:0004008), “electron transfer activity” (GO:0009055), and “copper ion transmembrane transporter activity” (GO:0005375). There were 4 common significantly enriched GO terms in the above three pairwise comparisons, including “sequence-specific DNA binding” (GO:0043565), “DNA repair” (GO:0006281), “transcription regulatory region DNA binding” (GO:0044212), and “cell differentiation” (GO:0030154). The 30 most significant terms in the GO enrichment analysis of the predicted target genes are shown in Supplementary Figure 9.

To learn more about how miRNAs and target genes are regulated during continuous cropping, KEGG annotation was performed on all DEMI target genes (Supplementary Figure 10). In SP_S2 vs. FP_S2, “Riboflavin metabolism” (ko00740) and “Purine metabolism” (ko00230) contained the greatest number of target genes, followed by “Glycerolipid metabolism” (ko00561), “Phosphatidylinositol signaling system” (ko04070), and “Glycerophospholipid metabolism” (ko00564). In SP_S3 vs. FP_S3, “Peroxisome” (ko04146) contained the greatest number of target genes, followed by “Spliceosome” (ko03040) and “Amino sugar and nucleotide sugar metabolism” (ko00520). There were only two pathways in SP_ S4 vs. FP_ S4 that were significantly enriched as: “MAPK signaling pathway - plant” (ko04016) and “ubiquitin mediated proteolysis” (ko04120).

### Combined Expression Analysis of miRNAs and Their Target Genes

**Figure 4:**
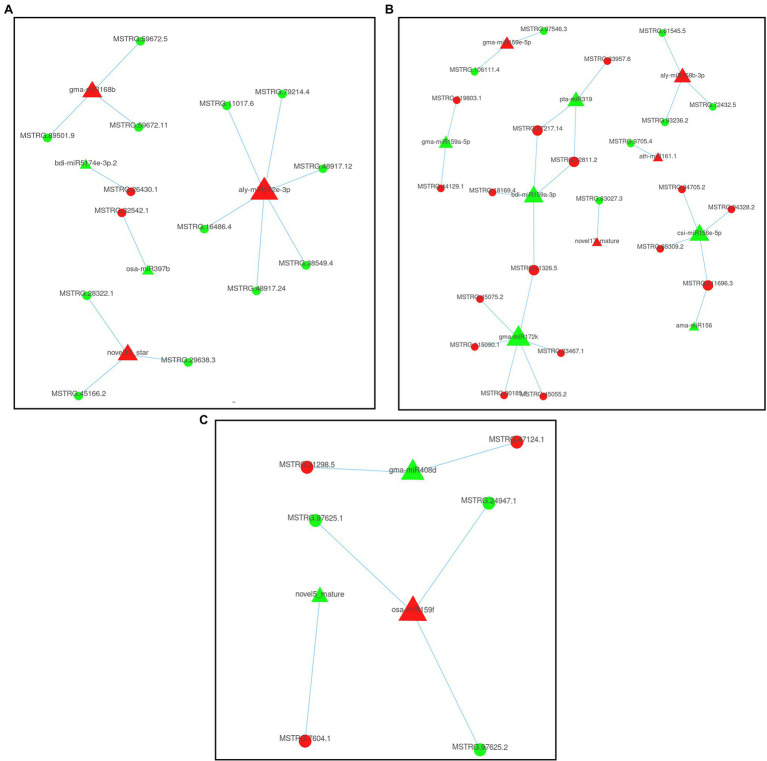
Potential miRNA and mRNA regulatory network of patchouli in response to continuous cropping obstacle. **(A)** SP_S2 vs. FP_S2; **(B)** SP_S3 vs. FP_S3; and **(C)** SP_S4 vs. FP_S4.

**Figure 5:**
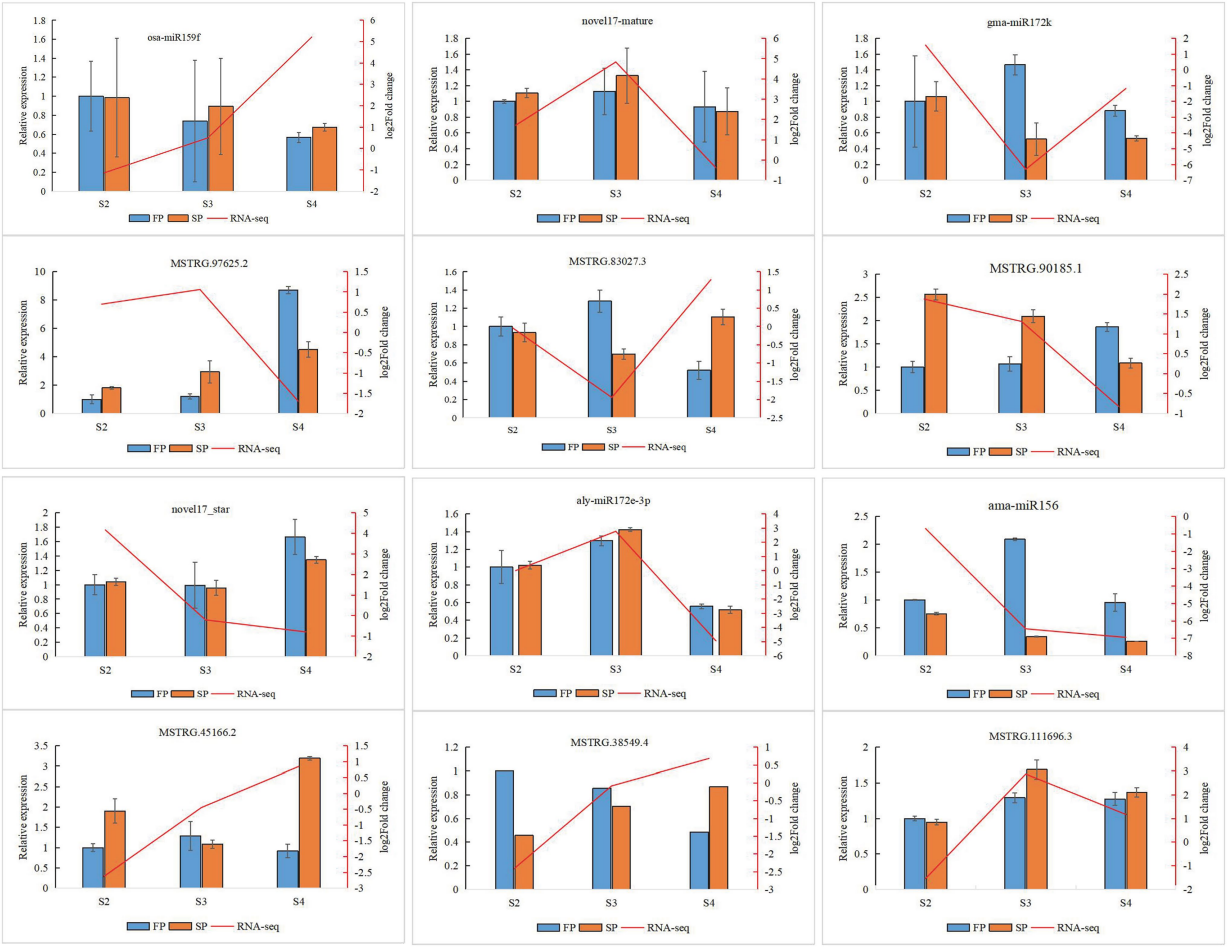
Overview of miRNAs and genes regulation patterns in the patchouli response to continuous cropping. The red letters are upregulated genes or miRNAs, and the blue letters are downregulated genes or miRNAs. “⊥” stands for suppression.

To explore the regulatory relationship between miRNAs and genes under continuous cropping conditions, we constructed a regulatory network diagram of DEMIs and DEGs (Figure 4). Eighteen DEMIs negatively regulated the expression of 43 target genes (DEGs). A single miRNA can regulate multiple target genes, as shown in Figure 4, while a single target gene can also be targeted by multiple miRNAs. In the S2 stage, five DEMIs negatively regulated the expression of 14 target genes, including 2 upregulated and 12 downregulated DEGs (Figure 4A). Ten DEMIs negatively regulated the expression of 23 target genes, and 3 DEMIs negatively regulated the expression of 6 target genes in the S3 (Figure 4B) and S4 (Figure 4C) stages. Homologous annotation of these 43 target genes revealed that they play significant roles in functions such as defense response, protein transport, root growth, signal transduction, and RNA synthesis (Figure 5; Supplementary Table 8). Among the 47 miRNA-mRNA pairs, eight pairs negatively regulate genes encoding TFs. For example, aly-mir172e-3p and gma-mir172k negatively regulate the expression of the AP2 gene, gma-mir172k and bdi-mir159a-3p negatively regulate the expression of the AP2-1 gene, ama-mir156 and csi-mir156e-5p negatively regulate the expression of the SPL3 gene, and osa-mir159f negatively regulates the expression of two GAMYB genes. These results indicated that these negative-regulatory miRNA-mRNA interaction pairs were involved in key biological processes such as signal transduction, plant development, and stress response.

### Validation of Gene Expression by qRT-PCR

**Figure 6:**
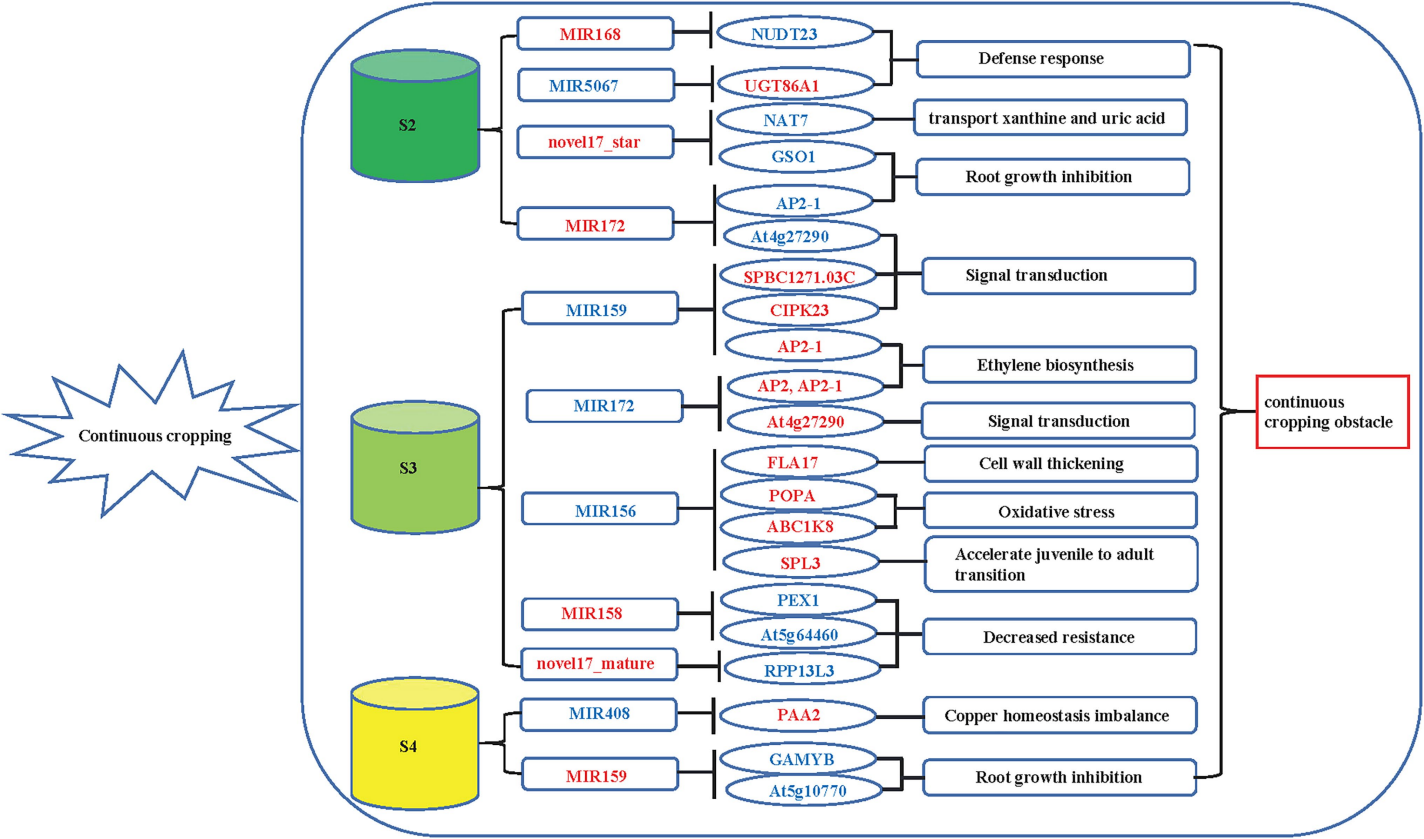
Quantitative real-time PCR (qRT-PCR) analysis of DEGs. The error bar represents the error values of three biological replicates.

**Figure 7:**
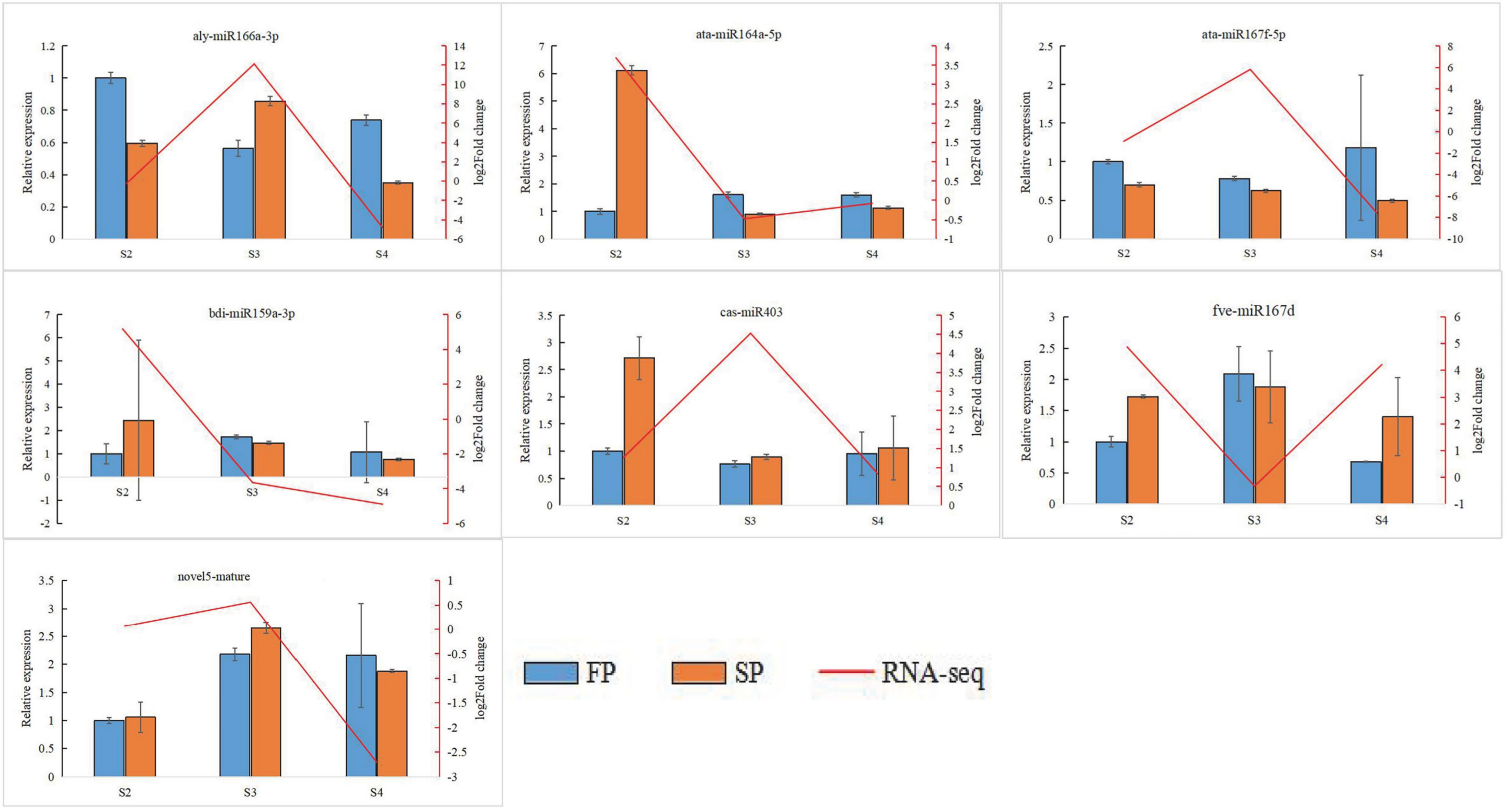
Quantitative real-time PCR analysis of DEMIs. The error bar represents the error values of three biological replicates.

**Figure 8:**
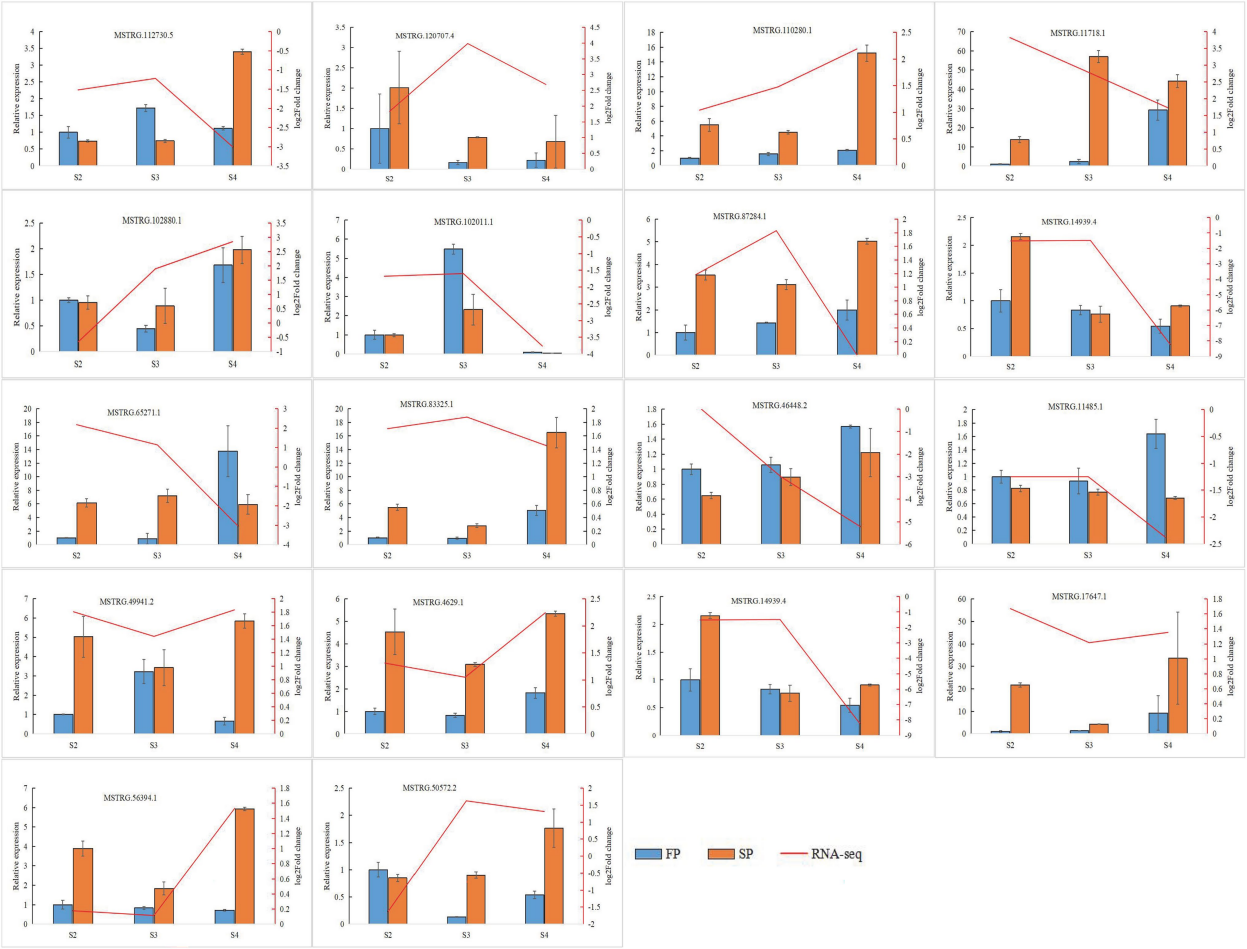
Quantitative real-time PCR validation of six identified key miRNA-mRNA interaction pairs for patchouli in response to continuous cropping. Six identified key miRNA-mRNA interaction pairs are osa-miR159f-MSTRG.97625.2, novel17_mature-MSTRG.83027.3, gma-miR172k-MSTRG.90185.1, novel17_star-MSTRG.45166.2, aly-miR172e-3p-MSTRG.38549.4, and ama-miR156-MSTRG.111696.3. The error bar represents the error values of three biological replicates.

Quantitative real-time PCR was used to validate the expression of 18 randomly selected DEGs and seven DEMIs in all 18 samples. qRT-PCR analysis of most of the samples showed that the expression trends of DEGs (Figure 6) and DEMIs (Figure 7) were similar to those observed by HTS. These findings show that the results of HTS and qRT-PCR were very similar. Additionally, to further verify the reliability of these miRNA-mRNA pairs related to continuous cropping response, six pairs of miRNA-mRNA were selected for qRT-PCR analysis (osa-miR159f-MSTRG.97625.2, novel17_mature-MSTRG.83027.3, gma-miR172k-MSTRG.90185.1, novel17_star-MSTRG.45166. 2, aly-miR172e-3p-MSTRG.38549.4, and ama-miR156-MSTRG.111696.3). The expression of these miRNAs was significantly negatively correlated with the expression of their corresponding target genes (Figure 8).

## Discussion

Continuous cropping obstacles have become a crucial factor restricting the improvement of yield and quality of many medicinal crops (Zhang et al., 2020). Approximately, 70% of medicinal plants using roots or rhizomes as medicine have different degrees of continuous cropping disorder obstacles in the cultivation process (Zhang and Lin, 2009). Therefore, studying the response mechanism of crops under continuous cropping in detail is critical. The crop response to continuous cropping is complex and involves multiple pathways. The rapid development of next-generation sequencing technology has facilitated the study of complex molecular mechanisms of the crop response to continuous cropping. In this study, the dynamic expression patterns of miRNA and mRNA in the roots of continuous cropping and noncontinuous cropping patchouli at different growth periods were evaluated to reveal the response mechanism of patchouli roots to continuous cropping obstacles. Patchouli continuous cropping could significantly inhibit the growth and development of roots, inhibiting the growth of plants and reducing the yield. Additionally, under continuous cropping stress, patchouli roots activate or start the expression of cell specific miRNA, change the expression program of normal genes, cause the adjustment and response of the intracellular core metabolic pathway, and lead to poor growth and even death of plants. These findings shed fresh light on the patchouli continuous cropping response mechanism. Next, we will focus on the crucial results obtained in this experiment.

### Continuous Cropping Significantly Changes the Physiological and Biochemical Characteristics of Patchouli Roots

Many studies have shown that continuous cropping has effects on plant morphological, physiological, biochemical, and molecular characteristics (He et al., 2019; Wang et al., 2020). During the patchouli planting process, we observed root browning, branch reduction, and low root activity under continuous cropping. This phenomenon led directly to a decline in the ability of patchouli roots to absorb nutrients and water under continuous cropping, thereby reducing crop yield and quality. Furthermore, studies have revealed that continuous cropping aggravates the degree of cell membrane lipid peroxidation, enhances or inhibits the activities of the plant protective enzymes POD, SOD, and CAT, increases the level of MDA, and damages the normal structure and function of the membrane (Zhang et al., 2010, 2011; Dong et al., 2018). In this study, we examined the influence of continuous cropping on some physiological and biochemical characteristics of patchouli. MDA is one of the peroxidation products produced by intracellular reactive oxygen species (ROS) attacking the membrane lipid system, and its level is often used to evaluate the degree of membrane lipid peroxidation and cell injury (Ma et al., 2015). Compared with FP, SP had a higher MDA content, suggesting a higher level of continuous cropping-induced lipid peroxidation in patchouli roots. In response to the exacerbation of membrane lipid peroxidation and cell membrane damage caused by ROS accumulation, plants have developed a set of cellular enzymatic defense systems consisting of POD, SOD, and CAT (Wang et al., 2004). These systems play an important role in eliminating intracellular ROS and maintaining the dynamic balance of ROS. In this study, continuous cropping significantly enhanced the activities of POD, SOD, and CAT in patchouli roots. The results suggested that short-term continuous cropping would prompt plants to initiate protective enzyme systems to defend against and scavenge free radicals and protect cells from oxidative damage, which is similar to previous findings in *A. paniculata* (Li J. et al., 2017). Soluble sugars and soluble proteins are important osmoregulatory substances that are closely related to the degree of injury and resistance of plants under stress (Yang et al., 2020). Free proline is considered to be a compatible penetrant that contributes to crop resistance to osmotic stress. Under stress conditions, the mass ratio of proline in plant cells greatly increases, thereby reducing cell osmotic potential, helping cells absorb water, and preventing dehydration of the protoplasm and protein molecules (Bao et al., 2020). In this study, it was found that continuous cropping increased the levels of osmoregulatory substances (Pro, SS, and SPr). Our results were in accordance with previous studies on cotton (Zhang Y. et al., 2016), which also reported that the content of osmoregulatory substances increased significantly under continuous cropping conditions. The phenotypic and physiological characteristics showed that continuous cropping caused serious harm to the growth and development of patchouli.

### Transcriptomic Differences in Patchouli in Response to Continuous Cropping Obstacles

Continuous cropping obstacles are a common disadvantage of modern agricultural practices, especially for medicinal crops (Wu and Lin, 2020). Continuous cropping has become a key factor restricting the quality and development of medicinal crops. Many studies have shown that soil properties, rhizosphere microbial community structure, and allelopathy are the main causes of continuous cropping obstacles (Xin et al., 2019; Zhang et al., 2020). The first step to understanding the mechanism underlying the development of continuous cropping obstacles is to determine how plants perceive the harmful signals produced by continuous cropping (Li M. et al., 2017). Recent advances in genome sequencing, assembly, and annotation have facilitated research on many crop traits, including stress tolerance. To cope with different environmental stress conditions, plants exhibit specific changes in gene expression, metabolism, and physiology. It is helpful to identify stress-responsive genes by comparing gene expression differences between normal and stressed plants. Continuous cropping obstacles actually represent a kind of stress. The use of HTS technology to analyze the molecular mechanisms of medicinal crops in response to continuous cropping has attracted the attention of an increasing number of researchers. For example, Dong et al. (2018) analyzed the transcriptome and proteome of *Nelumbo nucifera* under continuous cropping to research the potential molecular mechanism underlying the response to continuous cropping. He et al. (2019) found 762 DEGs in the leaves of *Codonopsis tangshen* under continuous cropping using the RNA-seq method, and these DEGs were mainly involved in the “tyrosine degradation I,” “glycogen synthesis,” “phenylalanine and tyrosine catabolism,” “signal transduction,” and “immune system” metabolic pathways. Li et al. (2019) identified, through transcriptome sequencing, 6,193 unigenes that were significantly differentially expressed in *A. paniculata* after succession cropping. Yang et al. (2013) constructed a transcriptome library of *R. glutinosa* roots and found a set of key genes responding to replanting-related disease using RNA-seq and digital gene expression profiling (DGE) technology. These studies provide research insight and approaches to further elucidate the molecular mechanisms by which plants respond to continuous cropping obstacles.

In the present study, we observed that continuous cropping severely endangers the growth and development of patchouli. This study revealed a difference in gene expression in response to continuous cropping in patchouli. Eighteen root cDNA libraries for the three growth periods of FP and SP patchouli plants were constructed and sequenced using the Illumina HiSeq X Ten platform. Compared with FP and SP in the same period, a total of 28,208 DEGs were identified in the three periods. The number of DEGs increased with increasing growth period duration, and the greatest number of DEGs was observed in the S4 period, indicating that more complex molecular mechanisms occurred in the late period of continuous cropping and that transcriptional regulation during this period was more complex. This is consistent with our physiological analysis results showing obvious differences in root morphology and various physiological indexes in the late growth period of SP compared with FP. GO enrichment analysis of DEGs showed that the DEGs were mainly enriched in cell, cell parts, organelles, cell processes, metabolic processes, response to stimuli, binding, catalytic activity, and transporter activity. However, the significantly enriched GO terms differed among periods, indicating that the response mechanisms to continuous cropping differed among periods. Likewise, the significantly enriched pathways also differed among periods. DEGs are mainly enriched in Metabolism, Genetic Information Processing, and Environmental Information Processing.

In S2 period, the upregulated DEGs after continuous cropping were mainly significantly enriched in plant-pathogen interaction, plant hormone signal transduction, glycolysis/gluconeogenesis, and protein processing in endoplasmic reticulum pathways. Genes encoding Ca^2+^/calmodulin-dependent protein kinase (CCaMK), E3 ubiquitin-protein ligase, ethylene receptor, serine/threonine-protein kinase, and abscisic acid receptor were significantly upregulated in these pathways. As a decoder of Ca^2+^ signaling, CCaMK is involved in plant responses to abiotic stress (Chen et al., 2021). Previous studies have shown that rice CCaMK OsDMI3 is a positive regulator of ABA responses, including seed germination, root growth, antioxidant defense, and tolerance to water and oxidative stress (Shi et al., 2012, 2014; Ni et al., 2019). In the present study, CCaMK, MAPK, and abscisic acid receptor were significantly upregulated in patchouli root after continuous cropping. Similarly, genes encoding Ca^2+^ signaling, MAPK and ethylene signaling, and chromatin modification were all specifically upregulated in continuous cropping *R. glutinosa* (Yang et al., 2015; Gu et al., 2020). From these findings, we speculate that the Ca^2+^ signal in the root of patchouli is rapidly activated after continuous cropping and acts on the upstream of MAPK to activate the antioxidant defense system in the ABA signal to resist continuous stress. By contrast, downregulated DEGs were mainly enriched in protein processing in endoplasmic reticulum, galactose metabolism, and spliceosome. Among these pathways, some genes encoding heat shock protein, stachyose synthase, and serine/arginine-rich splicing factor were significantly downregulated in continuous cropping patchouli. During the S3 period, some genes in the mRNA surveillance pathway, glycerolipid metabolism pathway, alanine, aspartate, and glutamate metabolism, and glutathione pathway were significantly upregulated in continuous patchouli root, such as genes encoding serine/threonine-protein phosphatase and diacylglycerol kinase. Many studies have confirmed that diacylglycerol kinase can play a critical role in plant growth and development and stress tolerance through the phosphatidic acid (PA) signaling pathway (Kue Foka et al., 2020). By contrast, some genes, such as those encoding glucose-1-phosphate adenylyltransferase, isoamylase, sucrose-phosphate synthase, myo-inositol-1-phosphate synthase, phosphatidylinositol-3-phosphatase, and phosphoinositide phosphatase, were significantly downregulated in continuous cropping patchouli. This finding indicates that continuous cropping stress may inhibit the expression of genes related to energy synthesis in the roots of patchouli, inhibiting the growth and development of the roots of patchouli. In the S4 period, we found that genes encoding heat shock cognate protein, calcium-binding protein, serine/threonine-protein kinase, and mitogen-activated protein kinase were significantly upregulated. With the extension of continuous cropping time, the related genes involved in the MAPK (mitogen-activated protein kinase) cascade signaling pathway in the roots of SP were gradually activated, particularly MPK9 and MPK18. The MAPK cascade pathway has been proven to play an essential role in the signal transduction of biotic and abiotic stresses and during plant development (Xu and Zhang, 2015; Sözen et al., 2020). Additionally, no significant difference was found in the expression of ethylene synthesis-related genes in the early stage of continuous cropping. However, with the increased stress time, the expression in the roots of continuous cropping patchouli was significantly higher than that of noncontinuous cropping. This finding is consistent with the results detected in the root transcriptome of *R. glutinosa* (Yang et al., 2015). Studies have demonstrated that activation of ethylene signaling leads to excessive accumulation of ethylene, which inhibits root growth and accelerates plant senescence (Iqbal et al., 2017). Generally, after patchouli root was subjected to continuous cropping stress, the stress signal was sensed and transmitted through the Ca^2+^ signal and MAPK signal in the cell to activate the plant stress resistance process. This response in turn induces the synthesis of ethylene, which accelerates the aging process and cell death. These phenomena block core metabolic pathways in patchouli, disrupting the program of gene regulation and enabling genes that should not normally be turned on.

Additionally, patchouli is a valuable medicinal plant rich in patchouli alcohol and pogostone. The continuous cropping of patchouli would result in a decreased content of patchouli essential oil and patchouli alcohol (He et al., 2017). In plants, patchouli alcohol comes from C5 unit isopentenyl diphosphate (IPP), which is through cytosolic mevalonate (MVA) and plastic 2-C Methyl-d-erythritol-4-phosphate (MEP) pathways (Yan et al., 2021a). Then, IPP generates dimethyl propylene pyrophosphate (DMAPP) under the action of isopentenyl pyrophosphate isomerase (IPI). IPP and DMAPP generated from the MVA or MEP pathway are catalyzed by farnesyl diphosphate synthase (FPS) to form farnesyl pyrophosphate (FPP) with a C15 skeleton, which is the common synthetic substrate of PA and other sesquiterpenoids. Finally, FPP is cycled by a different sesquiterpene synthase (TPS) to form different sesquiterpene carbon skeletons and then modified to form sesquiterpene with variable structures and functions. Previous studies have shown that 3-hydroxy-3-methylglutaryl coenzyme A reductase (HMGCR) and IPI are key rate-limiting enzymes of the MVA pathway and positively regulate the synthesis of patchouli alcohol (Zhang et al., 2019; Yan et al., 2021b). 1-Deoxy-D-xylulose-5-phosphate synthase (DXS), IPI (E)-4-hydroxy-3-methylbut-2-enyl-diphosphate synthase (IspG), and 4-hydroxy-3-methylbut-2-enyl-diphosphate reductase (IspH) have been reported to be key rate-limiting enzymes of the MEP pathway (Li Q. et al., 2017). On SP_S2-vs-FP_S2, five *IspH* genes were differentially expressed in the roots of FP and SP patchouli, and all were significantly downregulated in the roots of continuous cropping patchouli. One *IPI* gene was significantly upregulated in the roots of continuous cropping patchouli and one *DXS* gene was downregulated in the roots of continuous cropping patchouli. On SP_S3-vs-FP_S3, four *IspH* (two upregulated and two downregulated), one *IspG* (downregulated), and one *DXS* gene (upregulated) were differentially expressed in FP and SP patchouli roots. On SP_S4-vs-FP_S4, we found that eight *HMGCR* genes (five downregulated and three upregulated), one *DXS* (downregulated), one *IspH* (downregulated), and one *IPI* (downregulated) were differentially expressed in the roots of FP and SP patchouli. Furthermore, studies have shown that 1-deoxy-d-xylulose 5-phosphate reductoisomeras (*DXR*) is negatively correlated with the synthesis of patchouli alcohol (Ouyang et al., 2016). Three *DXR* genes were found to be differentially expressed in the three comparison groups, of which two were significantly upregulated and one was downregulated in continuous cropping patchouli. The results showed that the genes significantly related to the biosynthesis of patchouli alcohol in the roots of continuous cropping patchouli was inhibited, resulting in the decrease of patchouli alcohol accumulation in continuous cropping patchouli. After continuous cropping of medicinal plants such as *A. paniculata* (Li et al., 2019), *R. glutinosa* (Wu et al., 2015), and *Pseudostellariae heterophylla* (Chen et al., 2017), the active ingredients also decreased significantly.

Unfortunately, due to the specificity of plant species and the limitations of available databases, there remain many DEGs that have not been annotated and enriched. However, we demonstrated for the first time that continuous cropping induced/inhibited the expression of some patchouli genes, which may enrich the transcriptome data of plants. This study could also provide clear ideas for the response of other plant species to continuous cropping.

### TFs Involved in the Response to Continuous Cropping Obstacles

TFs have been confirmed to be involved in the regulation of various stress responses in plants. When plants are under stress, the genes encoding TFs display a rapid early response to the stress, and then, these early-response TFs drive changes in the expression of additional genes later in the stress response (Zhang et al., 2021). In this study, continuous cropping induced differential expression of genes encoding TFs. A total of 1,417 of 28,208 DEGs encoded TF family proteins. Among them, most of the DEGs encoding AP2/ERF-ERF and bHLH TFs were upregulated, whereas most of the DEGs encoding bZIP, MYB-related, HSF, GARP-G2-like, and HB-HD-ZIP TFs were downregulated. The AP2/ERF TF family is widely distributed in plants and is related to plant primary and secondary metabolic regulation, growth, and development, as well as responses to environmental stimuli. The AP2/ERF TF family contains four major subfamilies, namely, the AP2, RAV, DREB, and ERF subfamilies. Many ERF genes confer tolerance to a variety of biotic stresses when expressed ectopically in different kinds of plants. For instance, some ERFs activate the transcription of basic defense-related genes, pathogenesis-related (PR) genes, osmotin, chitinase, and β-1,3-glucanase (Licausi et al., 2013). Overexpression of CBF3 in Arabidopsis can induce the expression of genes encoding key enzymes for proline synthesis and promote proline synthesis, and the increase in the content of free proline can enhance cold tolerance in plants (Gilmour et al., 2000). Drought and heat can induce DREB2 gene expression, which in turn positively regulates downstream drought or heat stress-responsive genes (Lata and Prasad, 2011). In addition to directly regulating the response to biotic and abiotic stresses, AP2/ERFs can participate in the stress response through hormone signal transduction and hormone-mediated pathways (Xie et al., 2019). ERF71 can directly regulate the transcription of the lignin biosynthetic genes CCR1, ccr10, and C4H to induce radial root growth, alter root architecture, and improve drought tolerance (Lee et al., 2017; Li et al., 2018). In this study, we also identified DREB3, ERF071, RAP2-2, and CRF2, encoding AP2/ERF TFs, as being significantly differentially expressed in SP and FP. Consequently, we presumed that AP2/ERF TFs respond to continuous cropping stress *via* two stress-responsive modes, which are dependent on or independent of the hormonal signaling pathway.

WRKY TFs constitute one of the largest TF families in higher plants. This family plays a key role in the plant response to biotic and abiotic stimuli by regulating the plant hormone signal transduction pathway (Jiang et al., 2017). It has been reported that WRKY51 is a negative regulator of ethylene synthesis, which inhibits the formation of root hairs and adventitious roots (Hu et al., 2018). In this study, transcription profile analysis showed that two genes encoding WRKY51 were significantly upregulated in SP_S2, indicating that WRKY51 may inhibit ethylene synthesis and thus inhibit the root growth of patchouli under continuous cropping conditions. Wrky22 and WRKY33 also participate in the stress-induced defense response through the mitogen-activated protein kinase (MAPK) signaling pathway (Jiang et al., 2017). The transcription profile analysis in this study showed that nine genes encoding WRKY33 and 1 gene encoding WRKY22 were significantly downregulated in SP_S4, indicating that WRKY33 and WRKY22 may reduce the resistance of patchouli under continuous cropping conditions by inhibiting the MAPK pathway. Furthermore, Wang et al. (2015) identified 15 differentially expressed MYB genes (13 of which were significantly downregulated) involved in the response of *R. glutinosa* to continuous cropping. Likewise, we also found that 50 MYB genes were significantly downregulated in the three periods of continuous cropping. Recent research has indicated that numerous bZIP genes participate in biotic and abiotic stress responses in various plant species (Alves et al., 2013; Golldack et al., 2014). Similarly, bHLH TFs have also been reported to be involved in a variety of plant biotic and abiotic stress responses (Sun et al., 2018). In this study, we identified 90 DEGs encoding bZIPs and 84 DEGs encoding bHLHs from patchouli. Most genes encoding bHLHs were upregulated under continuous cropping stress, while most genes encoding bZIPs were downregulated under continuous cropping stress. These results suggest that TFs such as AP2/ERF, bZIP, bHLH, MYB, and WRKY TFs play an important role in the continuous cropping response of patchouli.

### Patchouli miRNAs Participate in Regulating the Response to Continuous Cropping Obstacles

Stress response-related transcriptome reprogramming also involves upregulation or downregulation of miRNAs, which leads to posttranscriptional gene silencing (Zhang et al., 2021). Plants induce the expression of specific miRNAs in response to different stresses, and it has been confirmed that certain miRNAs play important regulatory roles in plant adaptation to stress conditions. To identify miRNAs involved in specific responses to continuous cropping in *R. glutinosa*, Yang et al. (2011) determined the differential expression profile of miRNAs between FP and SP *R. glutinosa* by using HTS technology and preliminarily screened 32 miRNA families involved in the response to continuous cropping. Zhang H. et al. (2016) identified 39 DEMIs in FP and SP *S. miltiorrhiza* roots, and qRT-PCR experiments showed that five miRNAs negatively regulated the expression levels of seven target genes related to root development or stress responses. In this study, a total of 927 known miRNAs and 130 novel miRNAs were identified in the three growth periods of FP and SP patchouli roots, 747 of which belonged to 112 miRNA families. Among these miRNA families, MIR159, MIR166, MIR156, and MIR171 contained more than 50 miRNAs each. Of particular interest are the 28 families that were present in only SP. Some of these factors may therefore be involved in replanting-related disease.

In addition, we identified 67 significant DEMIs belonging to 24 miRNA families in three periods of SP and FP patchouli. MIR159 and MIR167 were differentially expressed in the three periods. The MIR167 family is one of the highly conserved miRNA families in plants and participates in regulating plant vegetative and reproductive organ development, flowering time, and stress responses, primarily by targeting ARF6, ARF8, and IAR3 (Liu et al., 2021). MIR159 activates gibberellin (GA)-responsive genes by negatively regulating GAMYB or GAMYB-like TFs (Achard et al., 2004). Many studies have shown that the MIR159-GAMYB-mediated regulatory module participates in the plant stress response (Medina et al., 2017; Gao et al., 2022). Therefore, we inferred that the MIR167-ARF and MIR159-GAMYB pathways played critical roles in the patchouli root response to continuous cropping. Target prediction and annotation indicated that these miRNAs played a role by regulating specific stress-responsive genes, such as genes encoding SPL, ARF, LAC, TCP, MYB, AP2, and HD-ZIP proteins. For example, MIR167 and MIR172 target ARF6, which is a positive regulator of adventitious rooting. In the S2 period, with an increase in the expression of MIR167 and MIR172 in patchouli under continuous cropping, the expression of ARF6 was positively regulated to promote adventitious rooting in response to the effect of continuous cropping stress on the root growth. Studies have shown that the miR156-SPLs regulation mode plays an important role in the biosynthesis of patchouli alcohol (Yu Z. et al., 2015). At the S3 stage, five miR156 were differentially expressed, of which three were upregulated in the roots of continuous cropping patchouli. On the contrary, three SPLs genes were significantly downregulated in continuous cropping patchouli. They may inhibit the biosynthesis of patchouli alcohol in continuous cropping patchouli. The MIR319/TCP pathways play critical roles in cell proliferation, leaf and flower shape, stem branching, and the biosynthesis and transport of JA and auxin, as well as in plant responses to abiotic stresses (Fang et al., 2021). LAC, a type of polyphenol oxidase that is widely present in higher plants, is a target gene of MIR397 and is mainly involved in lignin synthesis (Xu et al., 2019). Lignin in plants is the main component of secondary cell wall thickening and secondary growth. Lignin biosynthesis is not only the key adaptation for plant survival and water transport but also the key adaptation for plant defense. RNA-seq and miRNA-seq showed that the LAC gene was significantly upregulated and osa-miR397b was significantly downregulated in the roots of continuously cropped patchouli during the S2 and S3 periods. Upregulated expression of LAC accelerates the accumulation of lignin in roots and enables plant cell walls to resist the mechanical pressure of pathogen invasion. Further functional analysis showed that these target genes were mainly related to signal transduction, metabolism of cofactors and vitamins, nucleotide metabolism, carbohydrate metabolism, transport and catabolism, and transcription under continuous cropping obstacles.

### Potential miRNA Regulatory Network of Patchouli in Response to Continuous Cropping Obstacles

Plant miRNAs can respond to stress by targeting the negative regulation of specific target genes. Comprehensive analysis of miRNA and gene expression profiles will allow more accurate analysis of the interactions or regulatory mechanisms between miRNAs and mRNAs under stress conditions, thereby offering new perspectives for the study of stress resistance mechanisms in plants (Funikov and Zatcepina, 2017). In this study, joint analysis of miRNAs and mRNAs identified 47 miRNA-mRNA pairs involved in the response of patchouli to continuous cropping obstacles and allowed the construction of a regulatory network for these interactions. Based on homologous queries of these miRNAs, corresponding target genes found that these miRNA-mRNA pairs are involved in different aspects, most significantly the defense response, protein transport, root growth, signal transduction, and RNA synthesis. These findings suggested that these identified miRNA-mRNA pairs were the key miRNA-mRNA pairs related to the patchouli continuous cropping response.

MiR172 plays a role in the biotic stress response and root development through negative regulation of its target encoding the TF AP2 (Nova-Franco et al., 2015). In the current study, the expression of MIR172 was upregulated in the S2 period (downregulated in the S3 period), while that of its target encoding the AP2 TF was reversed, showing that MIR172 may be involved in root development under continuous cropping conditions. MIR156 targets the SPL TF; overexpression of this TF leads to increased lateral root production in *Arabidopsis thaliana*; however, when the MIR156 levels are increased, lateral root production decreases (Yu N. et al., 2015). Furthermore, Kim et al. (2012) showed that the miR156-SPL3 module in *Arabidopsis* regulates flowering in response to ambient temperature through FLOWERING LOCUS T. In this study, the expression of csi-miR156e-5p and ama-miR156 was downregulated, while that of the target gene encoding SPL was upregulated, suggesting that the MIR156-SPL regulatory module might promote the shift to reproductive growth under continuous cropping conditions. The pleiotropic effect of this response would be a reduction in vegetative and root growth. MIR159 negatively regulates the expression of GAMYB genes at the posttranscriptional level and may play critical roles in patchouli root development under continuous cropping conditions. In addition to the key TFs, the negative-regulatory modules were composed of numerous genes encoding essential enzymes or functional proteins, and miRNAs were also thought to play crucial roles in the continuous cropping obstacle response. The receptor-like kinases GASSHO1 (GSO1) and GSO2 together regulate root growth in *Arabidopsis* by controlling cell division and cell fate specification (Racolta et al., 2014). We found that the target mRNAs from novel17_ star-MSTRG.28322.1 pairs were homologous with GSO1, indicating that the gene might have a conserved regulatory role in the root growth of patchouli and the corresponding miRNAs were the key miRNAs for controlling growth. Novel17_star also negatively regulates a gene homologous to the encoding nucleobase-ascorbate transporter 7 (NAT7). Apple MdNAT7 may transport xanthine and uric acid to help scavenge ROS more effectively under salt stress (Sun et al., 2021). UGT86A1 (UDP glycosyltransferase 86A1) is a member of the glycosyltransferase (GT) family. GTs respond to various plant stresses by combining with various plant hormones and other metabolites. Liu et al. (2019) found that UGT86A1 and UGT88A1 in *Vitis pseudoeticulata* were significantly upregulated after inoculated into *Plasmapara viticola*. In this study, continuous cropping inhibited the expression of MIR5067 and upregulated the expression of its target gene UGT86A1 in the S2 period. MIR158 was activated in continuous cropping patchouli in the S3 period, inhibiting the expression of the genes PEX1 (encoding peroxisome biogenesis protein) and At5g64460 (encoding phosphoglycate mutase like protein), which are related to the defense response, resulting in the decline of stress resistance of continuous cropping patchouli. Additionally, we found that MIR172 negatively regulates At4g27290 encoding G-type lectin s-receptor-like serine/threonine-protein kinase, and MIR408d negatively regulates PAA2 encoding copper-transporting ATPase. These findings suggested that these miRNAs regulated the patchouli continuous cropping response through signal transduction pathways.

To further verify the reliability of these miRNA-mRNA pairs related to the continuous cropping response of patchouli, six miRNA-mRNA pairs involved in the defense response, protein transport, root growth, signal transduction, and flowering regulation were analyzed by qRT PCR. The expression of these miRNAs was significantly negatively correlated with their corresponding target genes. The above results show that these identified miRNA-mRNA pairs can be used as candidate miRNA-mRNA pairs in the patchouli continuous cropping response. Additionally, the combination of small RNA and transcriptome analysis is an effective method to identify key miRNAs in the patchouli continuous cropping response.

## Conclusion

This study is the first attempt to integrate the expression data of miRNA and mRNA to explore the response mechanism of patchouli root to continuous cropping during the formation of continuous cropping obstacles of patchouli. A total of 67 DEMIs and 28,208 DEGs were identified by RNA-seq. Through combined mRNA-miRNA analysis, 47 miRNA-target gene pairs related to defense responses, protein transport, root growth, signal transduction, RNA synthesis, and flowering regulation were identified. When patchouli was subjected to continuous cropping stress, the MAPK cascade signal and calcium ion signal in patchouli were activated, activating the expression of a series of downstream early-response genes. Phosphorylation of these early-response genes, such as those encoding Serine/threonine-protein kinase and/or receptor-like protein kinase, mediates programmed cell death and metabolic disorders and other pathological phenomena, disrupting the expression and function of normal genes, and resulting in poor plant growth and even death. During this period, continuous cropping also activated the expression of many specific miRNAs related to stress response and signal transduction, changed the expression program of genes related to normal plant growth and development, and caused the adjustment and response of core metabolic pathways.

## Data Availability Statement

The datasets presented in this study can be found in online repositories. The names of the repository/repositories and accession number(s) can be found at: https://www.ncbi.nlm.nih.gov/, PRJNA737065 https://www.ncbi.nlm.nih.gov/, PRJNA736767 https://www.ncbi.nlm.nih.gov/, PRJNA737190 https://www.ncbi.nlm.nih.gov/, PRJNA737191.

## Author Contributions

YW designed and supervised the project. WY, SC, ZY, CZ, and GY prepared the samples and implemented the experiment. WY, JY, and JZ analyzed the data. WY wrote the manuscript. YW and DY revised the manuscript. All authors contributed to the article and approved the submitted version.

## Funding

This work was financially supported by the High level Talents Program of Hainan Natural Science Foundation (no. 2019RC087), the National Natural Science Foundation of China (no. 81860681), and the open project of the Key Laboratory of Tropical Horticultural Crops Quality Control in Hainan Province (no. 202105).

## Conflict of Interest

The authors declare that the research was conducted in the absence of any commercial or financial relationships that could be construed as a potential conflict of interest.

## Publisher’s Note

All claims expressed in this article are solely those of the authors and do not necessarily represent those of their affiliated organizations, or those of the publisher, the editors and the reviewers. Any product that may be evaluated in this article, or claim that may be made by its manufacturer, is not guaranteed or endorsed by the publisher.

## Footnotes

^1^ http://www.sanger.ac.uk/software/Rfam

^2^ http://www.ncbi.nlm.nih.gov/genbank/

^3^ http://www.mirbase.org/

## References

[ref1] Achard P. Herr A. Baulcombe D. C. Harberd N. P. (2004). Modulation of floral development by a gibberellin-regulated microRNA. Development 131, 3357–3365. doi: 10.1242/dev.01206, PMID: 15226253

[ref2] Altschul S. F. Gish W. Miller W. Myers E. W. Lipman D. J. (1990). Basic local alignment search tool. J. Mol. Biol. 215, 403–410. doi: 10.1016/S0022-2836(05)80360-2 2231712

[ref3] Alves M. S. Dadalto S. P. Goncalves A. B. De Souza G. B. Barros V. A. Fietto L. G. (2013). Plant bZIP transcription factors responsive to pathogens: a review. Int. J. Mol. Sci. 14, 7815–7828. doi: 10.3390/ijms14047815, PMID: 23574941 PMC3645718

[ref4] Anders S. Huber W., (2012). Differential Expression of RNA-Seq Data at the Gene Level-the DESeq Package. Heidelberg, Germany: European Molecular Biology Laboratory (EMBL).

[ref5] Anders S. Pyl P. T. Huber W. (2015). HTSeq-a python framework to work with high throughput sequencing data. Bioinformatics 31, 166–169. doi: 10.1093/bioinformatics/btu638, PMID: 25260700 PMC4287950

[ref6] Bao G. Z. Tang W. Y. An Q. R. Liu Y. X. Zhao N. Zhu S. N. (2020). Physiological effects of the combined stresses of freezing-thawing, acid precipitation and deicing salt on alfalfa seedlings. BMC Plant Biol. 20:204. doi: 10.1186/s12870-020-02413-4, PMID: 32393175 PMC7216480

[ref7] Bolger A. M. Lohse M. Usadel B. (2014). Trimmomatic: a flexible trimmer for Illumina sequence data. Bioinformatics 30, 2114–2120. doi: 10.1093/bioinformatics/btu170, PMID: 24695404 PMC4103590

[ref8] Burkhardt A. Day B. (2016). Transcriptome and small RNAome dynamics during a resistant and susceptible interaction between cucumber and downy mildew. Plant Genome 9, 1–19. doi: 10.3835/plantgenome2015.08.0069, PMID: 27898768

[ref9] Chakrapani P. Venkatesh K. Singh B. C. S. Jyothi B. A. Kumar P. Amareshwari P.. (2013). Phytochemical, pharmacological importance of patchouli (*Pogostemon cablin* (Blanco) Benth) an aromatic medicinal plant. Int. J. Pharm. Sci. Rev. Res. 21, 7–15.

[ref10] Chen Y. Liu Z. Tu N. M. Hu Y. H. Jin C. Z. Luo Y. C.. (2020). Integrated transcriptome and microRNA profiles analysis reveals molecular mechanisms underlying the consecutive monoculture problem of *Polygonatum odoratum*. Cell. Mol. Biol. 66, 47–52. doi: 10.14715/2020.66.2.7, PMID: 32415926

[ref11] Chen M. Ni L. Chen J. Sun M. M. Qin C. H. Zhang G.. (2021). Rice calcium/calmodulin-dependent protein kinase directly phosphorylates a mitogen-activated protein kinase kinase to regulate abscisic acid responses. Plant Cell 33, 1790–1812. doi: 10.1093/plcell/koab071, PMID: 33630095 PMC8254507

[ref12] Chen J. Pan A. He S. J. Su P. Yuan X. L. Zhu S. W.. (2020). Different micro RNA families involved in regulating high temperature stress response during cotton (*Gossypium hirsutum* L.) anther development. Int. J. Mol. Sci. 21:1280. doi: 10.3390/ijms21041280, PMID: 32074966 PMC7072957

[ref13] Chen G. D. Wang L. Fabrice M. R. Tian Y. A. Qi K. J. Chen Q.. (2018). Physiological and nutritional responses of pear seedlings to nitrate concentrations. Front. Plant Sci. 9:1679. doi: 10.3389/fpls.2018.01679, PMID: 30515181 PMC6255940

[ref14] Chen J. Wu L. K. Xiao Z. G. Wu Y. H. Wu H. M. Qin X. J.. (2017). Assessment of the diversity of *Pseudomonas* spp. and *Fusarium* spp. in *Radix pseudostellariae* rhizosphere under monoculture by combining DGGE and quantitative PCR. Front. Microbiol. 8:1748. doi: 10.3389/fmicb.2017.01748, PMID: 28966607 PMC5605650

[ref15] Chen Y. Wu Y. G. Xu Y. Zhang J. F. Song X. Q. Zhu G. P.. (2014). Dynamic accumulation of sesquiterpenes in essential oil of *Pogostemon cablin*. Rev. Bras 24, 626–634. doi: 10.1016/j.bjp.2014.11.001

[ref16] Chen S. L. Yu H. Luo H. M. Wu Q. Li C. F. Steinmetz A. (2016). Conservation and sustainable use of medicinal plants: problems, progress, and prospects. Chin. Med. 11:37. doi: 10.1186/s13020-016-0108-7, PMID: 27478496 PMC4967523

[ref17] China Pharmacopoeia Committee (2015). Chinese Pharmacopoeia. Beijing, China: China Medica Science Press.

[ref18] Dong C. Wang R. Zheng X. F. Zheng X. W. Jin L. F. Wang H. J.. (2018). Integration of transcriptome and proteome analyses reveal molecular mechanisms for formation of replant disease in *Nelumbo nucifera*. RSC Adv. 8, 32574–32587. doi: 10.1039/C8RA06503A PMC9086348 35547670

[ref19] Fahlgren N. Carrington J. C. (2010). miRNA target prediction in plants. Methods Mol. Biol. 592, 51–57. doi: 10.1007/978-1-60327-005-2_4 19802588

[ref20] Fang Y. J. Zheng Y. Q. Lu W. Li J. Duan Y. J. Zhang S.. (2021). Roles of miR319-regulated TCPs in plant development and response to abiotic stress. Crop J. 9, 17–28. doi: 10.1016/j.cj.2020.07.007

[ref21] Friedländer M. R. Mackowiak S. D. Li N. Chen W. Nikolaus R. (2012). miRDeep2 accurately identifies known and hundreds of novel microRNA genes in seven animal clades. Nucleic Acids Res. 40, 37–52. doi: 10.1093/nar/gkr688, PMID: 21911355 PMC3245920

[ref22] Funikov S. Y. Zatcepina O. G. (2017). Regulation of microRNA activity in stress. Mol. Biol. 51, 496–505. doi: 10.1134/S0026893317030050, PMID: 28900074

[ref23] Gao X. Zhang Q. Zhao Y. Q. Yang J. He H. B. Jia G. X. (2022). The lre-miR159a-LrGAMYB pathway mediates resistance to grey mould infection in *Lilium regale*. Mol. Plant Pathol. 21, 749–760. doi: 10.1111/mpp.12923 PMC7214475 32319186

[ref24] Gilmour S. J. Sebolt A. M. Salazar M. P. Everard J. D. Thomashow M. F. (2000). Overexpression of the *Arabidopsis* CBF3 transcriptional activator mimics multiple biochemical changes associated with cold acclimation. Plant Physiol. 124, 1854–1865. doi: 10.1104/pp.124.4.1854, PMID: 11115899 PMC59880

[ref25] Golldack D. Li C. Mohan H. Probst N. (2014). Tolerance to drought and salt stress in plants: unraveling the signaling networks. Front. Plant Sci. 5:151. doi: 10.3389/fpls.2014.00151, PMID: 24795738 PMC4001066

[ref26] Griffiths-Jones S. Saini H. K. van Dongen S. Enright A. J. (2008). miRBase: tools for microRNA genomics. Nucleic Acids Res. 36(S1), D154–D158. doi: 10.1093/nar/gkm952 17991681 PMC2238936

[ref27] Gu L. Wu Y. F. Lin M. G. Yuan F. Y. Zhan S. Y. Wang F. J.. (2020). Identification of MAPK cascade genes response to consecutive monoculture stress in *Rehmannia glutinosa*. Int. J. Agric. Biol. 24, 591–602. doi: 10.17957/IJAB/15.1476

[ref28] Gutiérrez-García C. Ahmed S. S. S. J. Ramalingam S. Selvaraj D. Srivastava A. Paul S.. (2022). Identification of microRNAs from medicinal plant *Murraya koenigii* by high-throughput sequencing and their functional implications in secondary metabolite biosynthesis. Plan. Theory 11:46. doi: 10.3390/plants11010046 PMC8747174 35009050

[ref29] Hajyzadeh M. Turktas M. Khawar K. M. Unver T. (2015). miR408 overexpression causes increased drought tolerance in chickpea. Gene 555, 186–193. doi: 10.1016/j.gene.2014.11.002 25445265

[ref30] He Y. Peng F. Deng C. Xiong L. Huang Z. Y. Zhang R. Q.. (2018). Building an octaploid genome and transcriptome of the medicinal plant *Pogostemon cablin* from Lamiales. Sci. Data 5:180274. doi: 10.1038/sdata.2018.274, PMID: 30532075 PMC6289116

[ref31] He L. P. Wu Y. G. Zhang J. F. Hu X. W. (2017). Changes of volatile oil and patchouli alcohol contents of *Pogostemon cablin* under continuous cropping. J. Trop. Biol. 8, 169–173. doi: 10.15886/j.cnki.rdswxb.2017.02.007

[ref32] He Y. S. Zhang M. D. Zhou W. X. Ai L. Q. You J. W. Liu H. H.. (2019). Transcriptome analysis reveals novel insights into the continuous cropping induced response in *Codonopsis tangshen*, a medicinal herb. Plant Physiol. Biochem. 141, 279–290. doi: 10.1016/j.plaphy.2019.06.001, PMID: 31202192

[ref33] Hu Z. R. Wang R. Zheng M. Liu X. B. Meng F. Wu H. L.. (2018). TaWRKY 51 promotes lateral root formation through negative regulation of ethylene biosynthesis in wheat (*Triticum aestivum* L.). Plant J. 96, 372–388. doi: 10.1111/tpj.14038, PMID: 30044519

[ref34] Huang H. W. (2011). Plant diversity and conservation in China: planning astrategic bioresource for a sustainable future. Bot. J. Linn. Soc. 166, 282–300. doi: 10.1111/j.1095-8339.2011.01157.x 22059249

[ref35] Iqbal N. Khan N. A. Ferrante A. Trivellini A. Francini A. Khan M. I. R. (2017). Ethylene role in plant growth, development and senescence: interaction with other phytohormones. Front. Plant Sci. 8:475. doi: 10.3389/fpls.2017.00475 28421102 PMC5378820

[ref36] Jiang J. J. Ma S. H. Ye N. H. Jiang M. Cao J. S. Zhang J. H. (2017). WRKY transcription factors in plant responses to stresses. J. Integr. Plant Biol. 59, 86–101. doi: 10.1111/jipb.12513, PMID: 27995748

[ref37] Jung I. Kang H. Kim J. U. Chang H. Kim S. Jung W. (2018). The mRNA and miRNA transcriptomic landscape of *Panax ginseng* under the high ambient temperature. BMC Syst. Biol. 12(Suppl 2):27. doi: 10.1186/s12918-018-0548-z, PMID: 29560829 PMC5861484

[ref38] Kang Y. C. Yang X. Y. Liu Y. H. Shi M. F. Zhang W. N. Fan Y. L.. (2021). Integration of mRNA and miRNA analysis reveals the molecular mechanism of potato (*Solanum tuberosum* L.) response to alkali stress. Int. J. Biol. Macromol. 182, 938–949. doi: 10.1016/j.ijbiomac.2021.04.094, PMID: 33878362

[ref39] Kim D. Langmead B. Salzberg S. L. (2015). HISAT: a fast spliced aligner with low memory requirements. Nat. Methods 12, 357–360. doi: 10.1038/nmeth.3317, PMID: 25751142 PMC4655817

[ref40] Kim J. J. Lee J. H. Kim W. Jung H. S. Huijser P. Ahn J. H. (2012). The microRNA156-SQUAMOSA PROMOTER BINDING PROTEIN-LIKE3 module regulates ambient temperature-responsive flowering via FLOWERING LOCUS T in *Arabidopsis*. Plant Physiol. 159, 461–478. doi: 10.1104/pp.111.192369, PMID: 22427344 PMC3375978

[ref41] Kue Foka I. C. Ketehouli T. Zhou Y. Li X. W. Wang F. W. Li H. (2020). The emerging roles of diacylglycerol kinase (DGK) in plant stress tolerance, growth, and development. Agronomy 10:1375. doi: 10.3390/agronomy10091375

[ref42] Kumara M. Sinniah U. R. (2015). A comprehensive review on the phytochemical constituents and pharmacological activities of *Pogostemon cablin* Benth.: an aromatic medicinal plant of industrial importance. Molecules 20, 8521–8547. doi: 10.3390/molecules20058521, PMID: 25985355 PMC6272783

[ref43] Lata C. Prasad M. (2011). Role of DREBs in regulation of abiotic stress responses in plants. J. Exp. Bot. 62, 4731–4748. doi: 10.1093/jxb/err210, PMID: 21737415

[ref44] Lawrence B. M. (2009). A preliminary report on the world production of some selected essential oils and countries. Perfum. Flavor. 34, 38–44.

[ref45] Lee D. K. Yoon S. Kim Y. S. Kim J. K. (2017). Rice OsERF71- mediated root modification affects shoot drought tole-rance. Plant Signal. Behav. 12:e1268311. doi: 10.1080/15592324.2016.1268311, PMID: 27935412 PMC5289523

[ref46] Li J. R. Chen X. Z. Li S. M. Zuo Z. M. Zhan R. T. He R. (2020). Variations of rhizospheric soil microbial communities in response to continuous *Andrographis paniculata* cropping practices. Bot. Stud. 61:18. doi: 10.1186/s40529-020-00295-1, PMID: 32542518 PMC7295922

[ref47] Li J. R., Chen X. Z., Tang X. T., Zeng X. D., Zhuo Y. N., He R. (2017). Effects of continuous cropping on *Andrographis paniculata* growth and herb quality. Tradit. Chin. Drug Res. Clin. Pharmacol. 28, 797–801.

[ref48] Li J. R. Chen X. Z. Zhan R. T. He R. (2019). Transcriptome profiling reveals metabolic alteration in *Andrographis paniculata* in response to continuous cropping. Ind. Crop. Prod. 137, 585–596. doi: 10.1016/j.indcrop.2019.05.067

[ref49] Li Q. Y. Fan F. Y. Gao X. Yang C. Bi C. H. Tang J. L.. (2017). Balanced activation of IspG and IspH to eliminate MEP intermediate accumulation and improve isoprenoids production in *Escherichia coli*. Metab. Eng. 44, 13–21. doi: 10.1016/j.ymben.2017.08.005, PMID: 28864262

[ref50] Li J. J. Guo X. Zhang M. H. Wang X. Zhao Y. Yin Z. G.. (2018). OsERF71 confers drought tolerance via mo-dulating ABA signaling and proline biosynthesis. Plant Sci. 270, 131–139. doi: 10.1016/j.plantsci.2018.01.017, PMID: 29576066

[ref51] Li M. J. Yang Y. H. Chen X. J. Wang F. Q. Lin W. X. Yi Y. J.. (2013). Transcriptome-wide identification of *R. glutinosa* miRNAs and their targets: the role of miRNA activity in the replanting disease. PLoS One 8:e68531. doi: 10.1371/journal.pone.0068531, PMID: 23861915 PMC3702588

[ref52] Li M. J. Yang Y. H. Feng F. J. Zhang B. Chen S. Q. Yang C. Y.. (2017). Differential proteomic analysis of replanted *Rehmannia glutinosa* roots by iTRAQ reveals molecular mechanisms for formation of replant disease. BMC Plant Biol. 17:116. doi: 10.1186/s12870-017-1060-0, PMID: 28693420 PMC5504617

[ref53] Licausi F. Ohme-Takagi M. Perata P. (2013). APETALA2/ethylene responsive factor (AP2/ERF) transcription factors: mediators of stress responses and developmental programs. New Phytol. 199, 639–649. doi: 10.1111/nph.12291, PMID: 24010138

[ref54] Liu Y. El-Kassaby Y. A. (2017). Regulatory crosstalk between microRNAs and hormone signalling cascades controls the variation on seed dormancy phenotype at *Arabidopsis thaliana* seed set. Plant Cell Rep. 36, 705–717. doi: 10.1007/s00299-017-2111-6, PMID: 28197719

[ref55] Liu X. Huang S. Xie H. T. (2021). Advances in the regulation of plant development and stress response by miR167. Front. Biosci. 26, 655–665. doi: 10.52586/4974, PMID: 34590474

[ref56] Liu R. Q. Weng K. Dou M. R. Chen T. T. Yin X. Li Z. Q.. (2019). Transcriptomic analysis of Chinese wild *Vitis pseudoreticulata* in response to *Plasmopara viticola*. Protoplasma 256, 1409–1424. doi: 10.1007/s00709-019-01387-x, PMID: 31115695

[ref57] Livak K. J. Schmittgen T. D. (2001). Analysis of relative gene expression data using real-time quantitative PCR and the 2^−ΔΔCT^ method. Methods 25, 402–408. doi: 10.1006/meth.2001.1262, PMID: 11846609

[ref58] Ma J. Du G. Li X. Zhang C. Y. Gao J. K. (2015). A major locus controlling malondialdehyde content under water stress is associated with fusarium crown rot resistance in wheat. Mol. Gen. Genomics. 290, 1955–1962. doi: 10.1007/s00438-015-1053-3 25939503

[ref59] Mao X. Z. Cai T. Olyarchuk J. G. Wei L. P. (2005). Automated genome annotation and pathway identification using the KEGG Orthology (KO) as a controlled vocabulary. Bioinformatics 21, 3787–3793. doi: 10.1093/bioinformatics/bti430, PMID: 15817693

[ref60] Medina C. da Rocha M. Magliano M. Ratpopoulo A. Revel B. Marteu N.. (2017). Characterization of microRNAs from *Arabidopsis* galls highlights a role for miR159 in the plant response to the root-knot nematode *Meloidogyne incognita*. New Phytol. 216, 882–896. doi: 10.1111/nph.14717, PMID: 28906559

[ref61] Ni L. Fu X. P. Zhang H. Li X. Cai X. Zhang P. P.. (2019). Abscisic acid inhibits rice protein phosphatase PP45 via H_2_O_2_ and relieves repression of the Ca^2+^/CaM-dependent protein kinase DMI3. Plant Cell 31, 128–152. doi: 10.1105/tpc.18.00506, PMID: 30538152 PMC6391686

[ref62] Nova-Franco B. Iniguez L. P. Valdes-Lopez O. Alvarado-Affantranger X. Leija A. Fuentes S. I.. (2015). The micro-RNA172c-APETALA2-1 node as a key regulator of the common bean-*Rhizobium etli* nitrogen fixation symbiosis. Plant Physiol. 168, 273–291. doi: 10.1104/pp.114.255547, PMID: 25739700 PMC4424015

[ref63] Ouyang P. Y. Liu Y. L. Wang Y. Mo X. L. Zeng S. H. (2016). Aging and/or tissue - specific regulation of patchoulol and pogostone in two *Pogostemon cablin* (Blanco) Benth. Cultivars. Physiol. Plant. 158, 272–283. doi: 10.1111/ppl.12466, PMID: 27167188

[ref64] Pagano L. Rossi R. Paesano L. Marmiroli N. Marmiroli M. (2021). miRNA regulation and stress adaptation in plants. Environ. Exp. Bot. 184:104369. doi: 10.1016/j.envexpbot.2020.104369, PMID: 35174506

[ref65] Racolta A. Bryan A. C. Tax F. E. (2014). The receptor-like kinases GSO1 and GSO2 together regulate root growth in *Arabidopsis* through control of cell division and cell fate specification. Dev. Dyn. 243, 257–278. doi: 10.1002/dvdy.24066, PMID: 24123341

[ref66] Sam G. J. Alex B. Mhairi M. Ajay K. Eddy S. R. (2003). Rfam: an RNA family database. Nucleic Acids Res. 31, 439–441. doi: 10.1093/nar/gkg006, PMID: 12520045 PMC165453

[ref67] Shi B. Ni L. Liu Y. Zhang A. Tan M. Jiang M. (2014). OsDMI3-mediated activation of OsMPK1 regulates the activities of antioxidant enzymes in abscisic acid signaling in rice. Plant Cell Environ. 37, 341–352. doi: 10.1111/pce.12154, PMID: 23777258

[ref68] Shi B. Ni L. Zhang A. Cao J. Zhang H. Qin T.. (2012). OsDMI3 is a novel component of abscisic acid signaling in the induction of antioxidant defense in leaves of rice. Mol. Plant 5, 1359–1374. doi: 10.1093/mp/sss068, PMID: 22869603

[ref69] Singh R. Singh M. Srinivas A. Rao E. V. S. P. Puttanna K. (2015). Assessment of organic and inorganic fertilizers for growth, yield and essential oil quality of industrially important plant patchouli (*Pogostemon cablin*) (Blanco) Benth. J. Essent. Oil-Bear. Plants 18, 1–10. doi: 10.1080/0972060X.2014.929043

[ref70] Sözen C. Schenk S. T. Boudsocq M. Chardin C. Almeida-Trapp M. Krapp A.. (2020). Wounding and insect feeding trigger two independent MAPK pathways with distinct regulation and kinetics. Plant Cell 32, 1988–2003. doi: 10.1105/tpc.19.00917, PMID: 32265268 PMC7268812

[ref71] Sun T. T. Pei T. T. Yang L. L. Zhang Z. J. Li M. J. Liu Y. R.. (2021). Exogenous application of xanthine and uric acid and nucleobase-ascorbate transporter MdNAT7 expression regulate salinity tolerance in apple. BMC Plant Biol. 21:52. doi: 10.1186/s12870-021-02831-y, PMID: 33468049 PMC7816448

[ref72] Sun X. Wang Y. Sui N. (2018). Transcriptional regulation of bHLH during plant response to stress. Biochem. Biophys. Res. Commun. 503, 397–401. doi: 10.1016/j.bbrc.2018.07.123, PMID: 30057319

[ref73] Swamy M. K. Mohanty S. K. Sinniah U. R. Maniyam A. (2015). Evaluation of patchouli (*Pogostemon cablin* Benth.) cultivars for growth, yield and quality parameters. J. Essent. Oil-Bear. Plants 18, 826–832. doi: 10.1080/0972060X.2015.1029989

[ref74] Swamy M. K. Sinniah U. R. (2016). Patchouli (*Pogostemon cablin* Benth.): botany, agrotechnology and biotechnological aspects. Ind. Crop. Prod. 87, 161–176. doi: 10.1016/j.indcrop.2016.04.032

[ref75] Tan Y. Cui Y. Li H. Kuang A. Li X. Wei Y.. (2017). Rhizospheric soil and root endogenous fungal diversity and composition in response to continuous *Panax notoginseng* cropping practices. Microbiol. Res. 194, 10–19. doi: 10.1016/j.micres.2016.09.009, PMID: 27938858

[ref76] Tong A. Z. Liu W. Liu Q. Xia G. Q. Zhu J. Y. (2021). Diversity and composition of the *Panax ginseng* rhizosphere microbiome in various cultivation modesand ages. BMC Microbiol. 21:18. doi: 10.1186/s12866-020-02081-2, PMID: 33419388 PMC7792351

[ref77] Trapnell C. Williams B. A. Pertea G. Mortazavi A. Kwan G. van Baren M. J.. (2010). Transcript assembly and quantification by RNA-Seq reveals unannotated transcripts and isoform switching during cell differentiation. Nat. Biotechnol. 28, 511–515. doi: 10.1038/nbt.1621, PMID: 20436464 PMC3146043

[ref78] Wang S. Dong L. Q. Luo Y. Y. Jia W. J. Qu Y. (2019). Characterization of rhizosphere microbial communities in continuous cropping maca (*Lepidium meyenii*) red soil, Yunnan, China. Arch. Agron. Soil Sci. 66, 805–818. doi: 10.1080/03650340.2019.1639155

[ref79] Wang F. Q. Suo Y. F. Wei H. Li M. J. Xie C. X. Wang L. N.. (2015). Identification and characterization of 40 isolated *Rehmannia glutinosa* MYB family genes and their expression profiles in response to shading and continuous cropping. Int. J. Mol. Sci. 16, 15009–15030. doi: 10.3390/ijms160715009, PMID: 26147429 PMC4519885

[ref80] Wang Y. S. Wang J. Yang Z. M. Lu B. Wang Q. Y. Li S. Q.. (2004). Salicylic acid modulates aluminum induced oxidative stress in roots of *Cassia tora L.* Bot. Stud. 46, 819–828. doi: 10.1111/j.1365-3180.2004.00411.x

[ref81] Wang Y. Z. Zhu S. Y. Liu T. M. Guo B. Li F. Bai X. H. (2020). Identification of the rhizospheric microbe and metabolites that led by the continuous cropping of ramie (*Boehmeria nivea* L. Gaud). Sci. Rep. 10:20408. doi: 10.1038/s41598-020-77475-3, PMID: 33230149 PMC7683709

[ref82] Wu Y. G. Guo Q. S. He J. C. Lin Y. F. Luo L. J. Liu G. D. (2010). Genetic diversity analysis among and within populations of *Pogostemon cablin* from China with ISSR and SRAP markers. Biochem. Syst. Ecol. 38, 63–72. doi: 10.1016/j.bse.2009.12.006

[ref83] Wu Y. G. Guo Q. S. Zheng H. Q. (2007). Textual research on history of introdution and herbal medicine of *Pogostemon cablin*. Chin. J. Chin. Mater. Med. 20, 2114–2117. 18306740

[ref84] Wu H. M. Lin W. X. (2020). A commentary and development perspective on the consecutive monoculture problems of medicinal plants. Chin. J. Eco-Agric. 28, 775–793. doi: 10.13930/j.cnki.cjea.190760

[ref85] Wu L. K. Wang J. Y. Huang W. M. Wu H. M. Chen J. Yang Y. Q.. (2015). Plantmicrobe rhizosphere interactions mediated by *Rehmannia glutinosa* root exudates under consecutive monoculture. Sci. Rep. 5:15871. doi: 10.1038/srep15871, PMID: 26515244 PMC4626807

[ref86] Xie Z. L. Nolan T. M. Jiang H. Yin Y. H. (2019). AP2/ERF transcription factor regulatory networks in hormone and abiotic stress responses in *Arabidopsis*. Front. Plant Sci. 10, 228. doi: 10.3389/fpls.2019.00228 30873200 PMC6403161

[ref87] Xin A. Y. Li X. Z. Jin H. Yang X. Y. Zhao R. M. Liu J. K.. (2019). The accumulation of reactive oxygen species in root tips caused by autotoxic allelochemicals-A significant factor for replant problem of *Angelica sinensis* (Oliv.) Diels. Ind. Crop. Prod. 138:111432. doi: 10.1016/j.indcrop.2019.05.081

[ref88] Xu Y. Wu Y. G. Chen Y. Zhang J. F. Song X. Q. Zhu G. P.. (2015). Autotoxicity in *Pogostemon cablin* and their allelochemicals. Rev. Bras 25, 117–123. doi: 10.1016/j.bjp.2015.02.003

[ref89] Xu J. Zhang S. Q. (2015). Mitogen-activated protein kinase cascades in signaling plant growth and development. Trends Plant Sci. 20, 56–64. doi: 10.1016/j.tplants.2014.10.001, PMID: 25457109

[ref90] Xu X. Y. Zhou Y. P. Wang B. Ding L. Wang Y. Luo L.. (2019). Genome-wide identification and characterization of laccase gene family in *Citrus sinensis*. Gene 689, 114–123. doi: 10.1016/j.gene.2018.12.015, PMID: 30576804

[ref91] Yan W. P. Yang Y. Z. Wu Y. G. Yu J. Zhang J. F. Yang D. M.. (2021a). Isopentenyl diphosphate isomerase (*IPI*) gene silencing negatively affects patchouli alcohol biosynthesis in *Pogostemon cablin*. Plant Mol. Biol. Report. 39, 557–565. doi: 10.1007/s11105-020-01269-0

[ref92] Yan W. P. Ye Z. C. Cao S. J. Yao G. L. Yu J. Yang D. M.. (2021b). Transcriptome analysis of two *Pogostemon cablin* chemotypes reveals genes related to patchouli alcohol biosynthesis. PeerJ 9:e12025. doi: 10.7717/peerj.12025, PMID: 34527441 PMC8403477

[ref93] Yang Y. H. Chen X. J. Chen J. Y. Xu H. X. Li J. Zhang Z. Y. (2011). Differential miRNA expression in *Rehmannia glutinosa* plants subjected to continuous cropping. BMC Plant Biol. 11:53. doi: 10.1186/1471-2229-11-53, PMID: 21439075 PMC3078876

[ref94] Yang W. Fan T. Hu X. Y. Cheng T. H. Zhang M. Y. (2017). Overexpressing osa-miR171c decreases salt stress tolerance in rice. Plant Biol. 60, 485–492. doi: 10.1007/s12374-017-0093-0

[ref95] Yang Y. H. Li M. J. Li X. Y. Chen X. J. Lin W. X. Zhang Z. Y. (2015). Transcriptome-wide identification of the genes responding to replanting disease in *Rehmannia glutinosa* L. roots. Mol. Biol. Rep. 42, 881–892. doi: 10.1007/s11033-014-3825-y, PMID: 25410878

[ref96] Yang Y. H. Li M. J. Yi Y. J. Li R. F. Li C. X. Yang H.. (2021). Integrated miRNA-mRNA analysis reveals the roles of miRNAs in the replanting benefit of *Achyranthes bidentata* roots. Sci. Rep. 11:1628. doi: 10.1038/s41598-021-81277-6, PMID: 33452468 PMC7810699

[ref97] Yang H. Wang T. Yu X. Wang X. (2020). Enhanced sugar accumulation and regulated plant hormone signalling genes contribute to cold tolerance in hypoploid *Saccharum spontaneum*. BMC Genomics 21:507. doi: 10.1186/s12864-020-06917-z, PMID: 32698760 PMC7376677

[ref98] Yang Y. H. Zhang Z. Y. Fan H. M. Zhao Y. D. Li M. J. Li J.. (2013). Construction and analysis of a different expression cDNA library in *Rehmannia glutinosa* plants subjected to continuous cropping. Acta Physiol. Plant. 35, 645–655. doi: 10.1007/s11738-012-1105-9

[ref99] Ye J. B. Zhang X. Tan J. P. Xu F. Cheng S. Y. Chen Z. X.. (2020). Global identification of *Ginkgo biloba* microRNAs and insight into their role in metabolism regulatory network of terpene trilactones by high-throughput sequencing and degradome analysis. Ind. Crop. Prod. 148:112289. doi: 10.1016/j.indcrop.2020.112289

[ref100] Young M. D. Wakefield M. J. Smyth G. K. Oshlack A. (2010). Gene ontology analysis for RNA-seq: accounting for selection bias. Genome Biol. 11:R14. doi: 10.1186/gb-2010-11-2-r14, PMID: 20132535 PMC2872874

[ref101] Yu Y. Jia T. R. Chen X. M. (2017). The ‘how’ and ‘where’ of plant microRNAs. New Phytol. 216, 1002–1017. doi: 10.1111/nph.14834, PMID: 29048752 PMC6040672

[ref102] Yu N. Niu Q. W. Ng K. H. Chua N. H. (2015). The role of miR156/SPLs modules in *Arabidopsis* lateral root development. Plant J. 83, 673–685. doi: 10.1111/tpj.12919, PMID: 26096676

[ref103] Yu Z. X. Wang L. J. Zhao B. Shan C. M. Zhang Y. H. Chen D. F.. (2015). Progressive regulation of sesquiterpene biosynthesis in *Arabidopsis* and patchouli (*Pogostemon cablin*) by the miR156-targeted SPL transcription factors. Mol. Plant 8, 98–110. doi: 10.1016/j.molp.2014.11.002, PMID: 25578275

[ref104] Zeng J. R. Liu J. Z. Lu C. H. Ou X. H. Luo K. K. Li C. M.. (2020). Intercropping with turmeric or ginger reduce the continuous cropping obstacles that affect *Pogostemon cablin* (patchouli). Front. Microbiol. 11:579719. doi: 10.3389/fmicb.2020.579719, PMID: 33133047 PMC7578394

[ref105] Zhang J. F. He L. P. Wu Y. G. Ma W. T. Chen H. Ye Z. C. (2018). Comparative proteomic analysis of *Pogostemon cablin* leaves after continuous cropping. Protein Expr. Purif. 152, 13–22. doi: 10.1016/j.pep.2018.07.004, PMID: 30017744

[ref106] Zhang H. H. Jin W. B. Zhu X. L. Liu L. He Z. G. Yang S. S.. (2016). Identification and characterization of *Salvia miltiorrhizain* miRNAs in response to replanting disease. PLoS One 11:e0159905. doi: 10.1371/journal.pone.0159905, PMID: 27483013 PMC4970794

[ref107] Zhang Z. Li G. Niu M. Fan H. Li J. Lin W. (2011). Responses of physiological ecology and quality evaluation of *Rehmannia gltinosa* in continuous cropping. Chin. J. Chin. Mater. Med. 36, 1133–1136. 21842635

[ref108] Zhang X. Q. Li K. C. Xing R. E. Liu S. Chen X. L. Yang H. Y.. (2018). miRNA and mRNA expression profiles reveal insight into chitosan-mediated regulation of plant growth. J. Agric. Food Chem. 66, 3810–3822. doi: 10.1021/acs.jafc.7b06081, PMID: 29584426

[ref109] Zhang Z. Y. Lin W. X. (2009). Continuous cropping obstacle and allelopathic autotoxicity of medicinal plants. Chin. J. Eco-Agric. 17, 189–196. doi: 10.3724/SP.J.1011.2009.00189

[ref110] Zhang Y. N. Wang X. X. Li X. G. Xu W. H. (2016). Effects of continuous cropping on physiological and biochemical resistance of cotton to *Fusarium* wilt. Acta Ecol. Sin. 36, 4456–4464.

[ref111] Zhang B. Weston L. A. Li M. J. Zhu X. C. Weston P. A. Feng F. J.. (2020). *Rehmannia glutinosa* replant issues: root exudate-rhizobiome interactions clearly influence replant success. Front. Microbiol. 11:1413. doi: 10.3389/fmicb.2020.01413, PMID: 32714307 PMC7344158

[ref701] Zhang G. X. Wu Y. G. Muhammad Z. U. H. Yang Y. Z. Yu J. Zhang J. F.. (2019). cDNA cloning, prokaryotic expression and functional analysis of 3-hydroxy-3-methylglutaryl coenzyme A reductase (HMGCR) in Pogostemon cablin. Protein Expres. Purif. 163:105454. doi: 10.1016/j.pep.2019.105454, PMID: 31301429

[ref112] Zhang J. Xue B. Y. Gai M. Z. Song S. L. Jia N. N. Sun H. M. (2017). Small RNA and transcriptome sequencing reveal a potential miRNA-mediated interaction network that functions during somatic embryogenesis in *Lilium pumilum* DC. Fisch. Front. Plant Sci. 8:566. doi: 10.3389/fpls.2017.00566, PMID: 28473835 PMC5397531

[ref113] Zhang X. H. Zhang E. H. Wang H. Z. Lang D. Y. (2010). Effects of continuous cropping obstacle on growth of *Angelica sinensis* and its mechanism. Chin. J. Chin. Mater. Med. 35, 1231–1234. doi: 10.4268/cjcmm20101003, PMID: 20707187

[ref114] Zhang H. M. Zhu J. H. Gong Z. Z. Zhu J. K. (2021). Abiotic stress responses in plants. Nat. Rev. Genet. 23, 104–119. doi: 10.1038/s41576-021-00413-0 34561623

[ref115] Zhao X. C. Yang G. Y. Liu X. Q. Yu Z. D. Peng S. B. (2020). Integrated analysis of seed microRNA and mRNA transcriptome reveals important functional genes and microRNA-targets in the process of walnut (*Juglans regia*) seed oil accumulation. Int. J. Mol. Sci. 21:9093. doi: 10.3390/ijms21239093, PMID: 33260456 PMC7731449

[ref116] Zhu C. Zhang S. T. Zhou C. Z. Chen L. Zaripov T. Zhan D. M.. (2020). Integrated transcriptome, microRNA, and phytochemical analyses reveal roles of phytohormone signal transduction and ABC transporters in flavor formation of oolong tea (*Camellia sinensis*) during solar withering. J. Agric. Food Chem. 68, 12749–12767. doi: 10.1021/acs.jafc.0c05750, PMID: 33112139

