# Supplementary material for: Integrated Analysis of Physiological, mRNA Sequencing, and miRNA Sequencing Data Reveals a Specific Mechanism for the Response to Continuous Cropping Obstacles in Pogostemon cablin Roots
Source: Front Plant Sci. 2022 Apr 1;13:853110. doi: 10.3389/fpls.2022.853110 (PMC9010791; doi:10.3389/fpls.2022.853110)
Supplement: Supplementary file 1 [file Data_Sheet_1.docx]

***Supplementary Material***

**
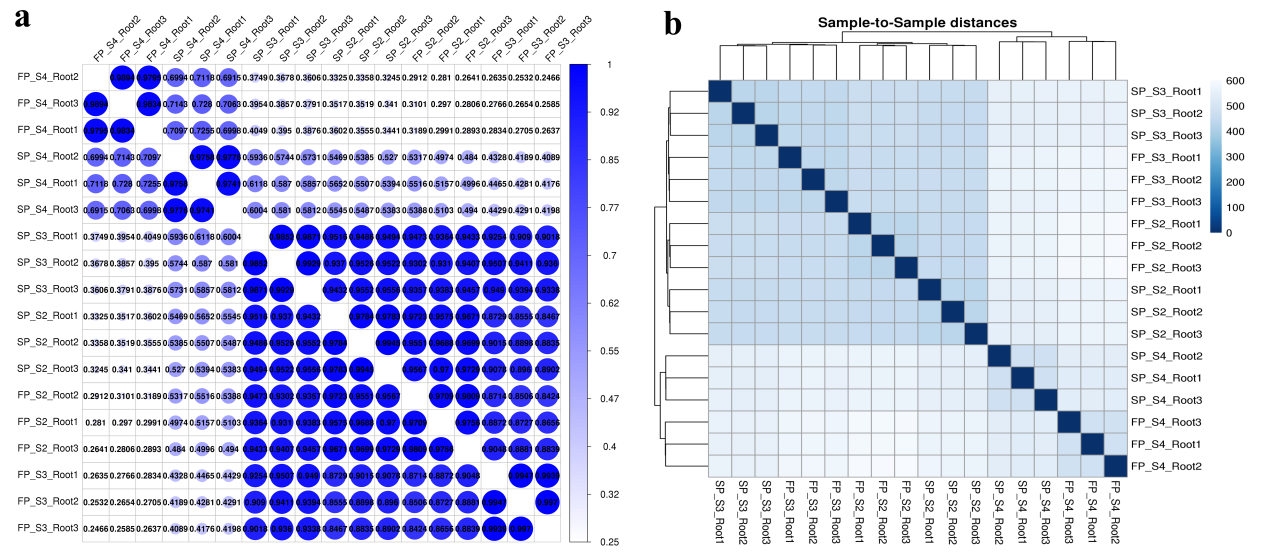
**

**Supplementary Figure 1. Correlation heat map (a) and cluster dendrogram (b)** **in transcriptomic profile of patchouli root samples.**

**
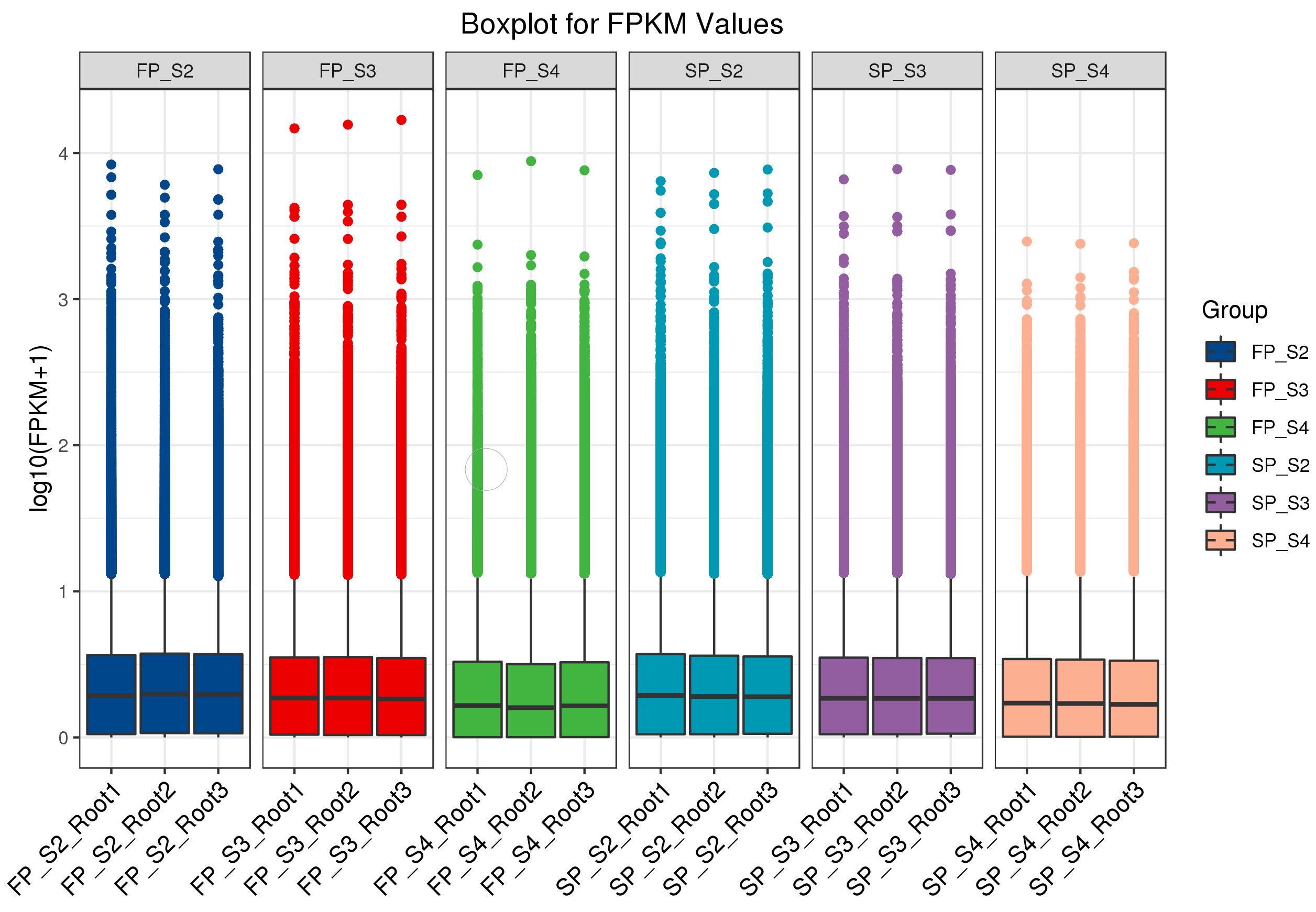
**

**Supplementary Figure 2. The fragments per kilobase of transcript per million mapped reads (FPKM) boxplot in the 18 libraries.**

**
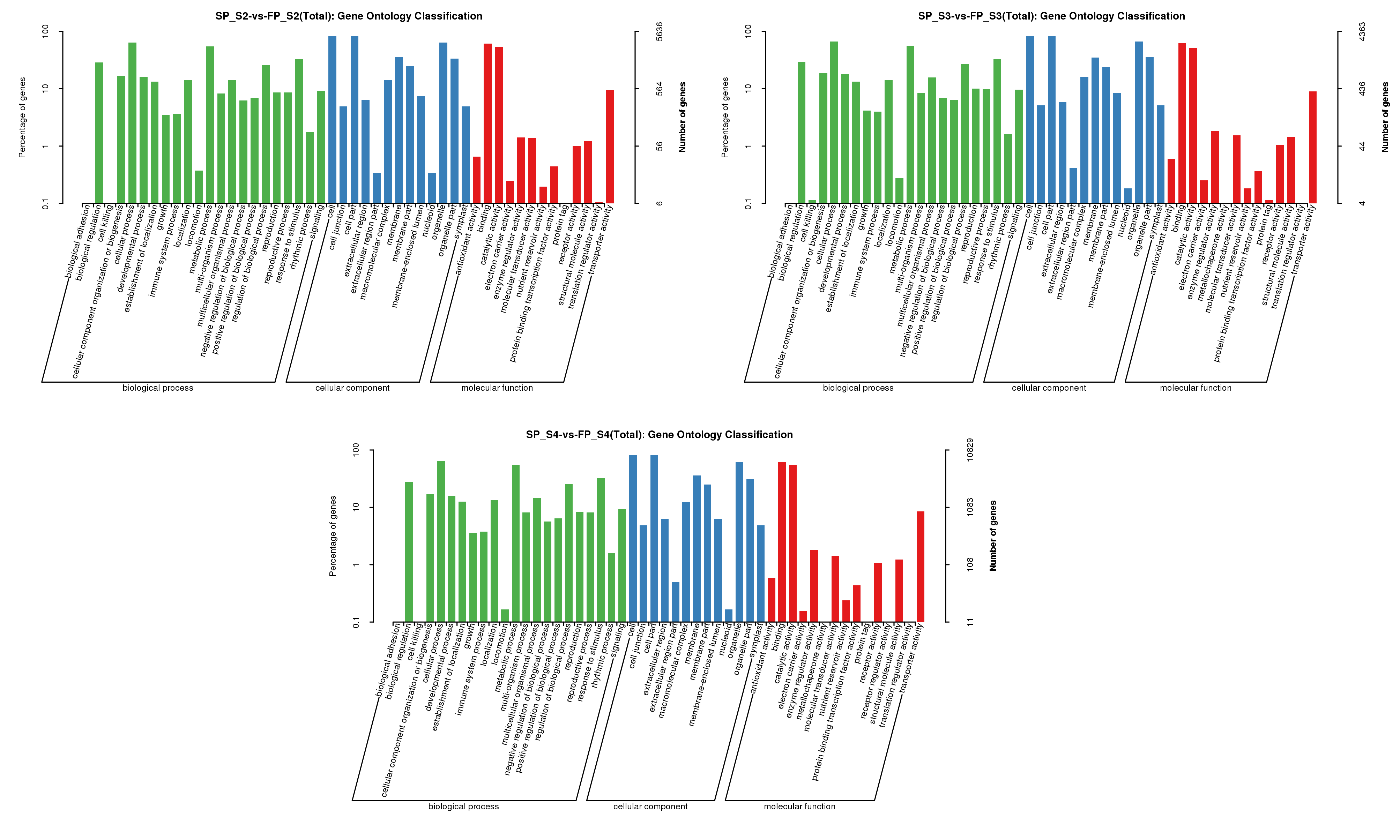
**

**Supplementary Figure 3. GO enrichment analysis of the DEGs** **in continuous cropping-treated root tissues against the controls.**

**
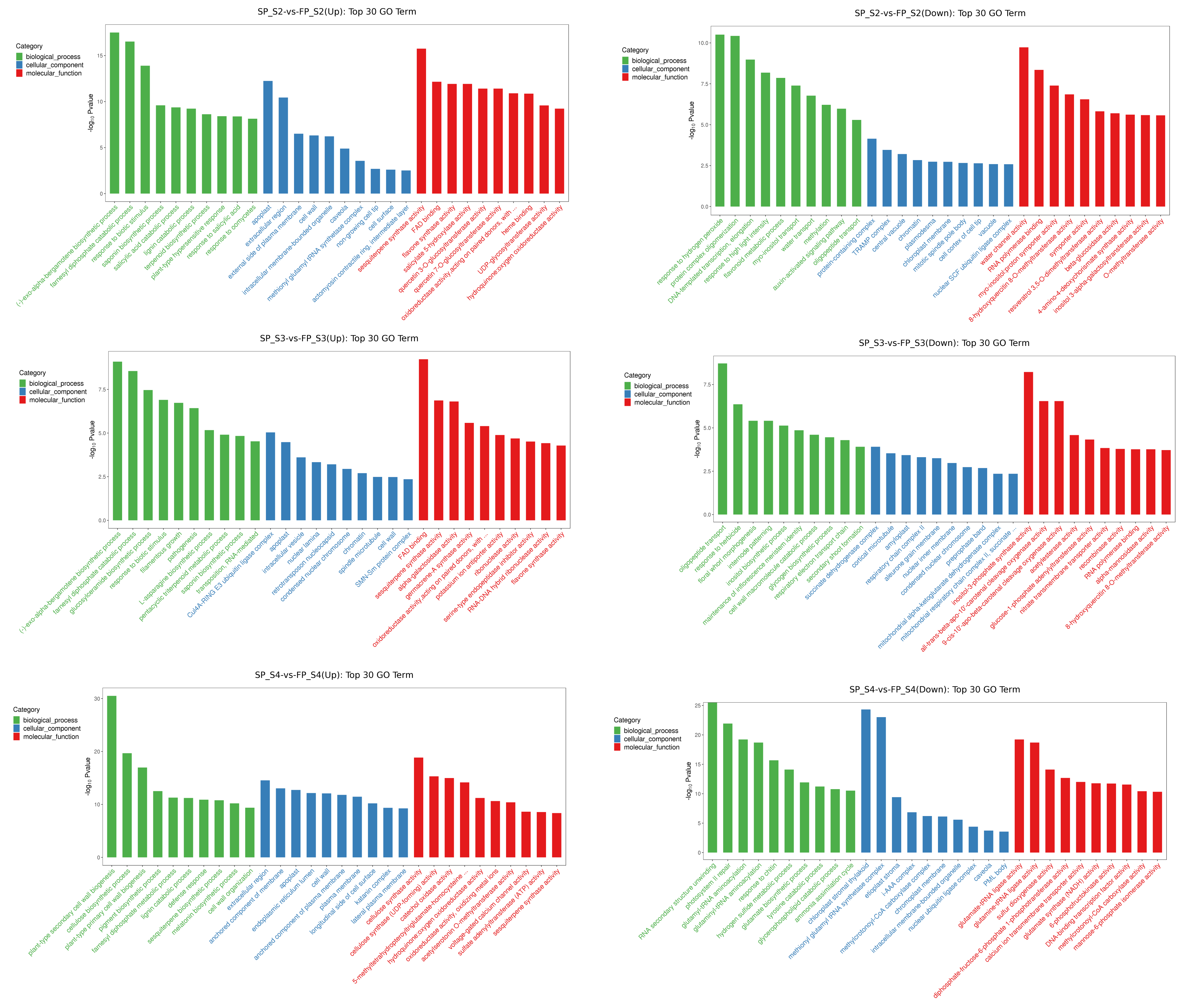
**

**Supplementary Figure 4. The 30 most significant terms in the GO enrichment analysis of up-and downregulated DEGs in continuous cropping-treated root tissues against the controls.**

**
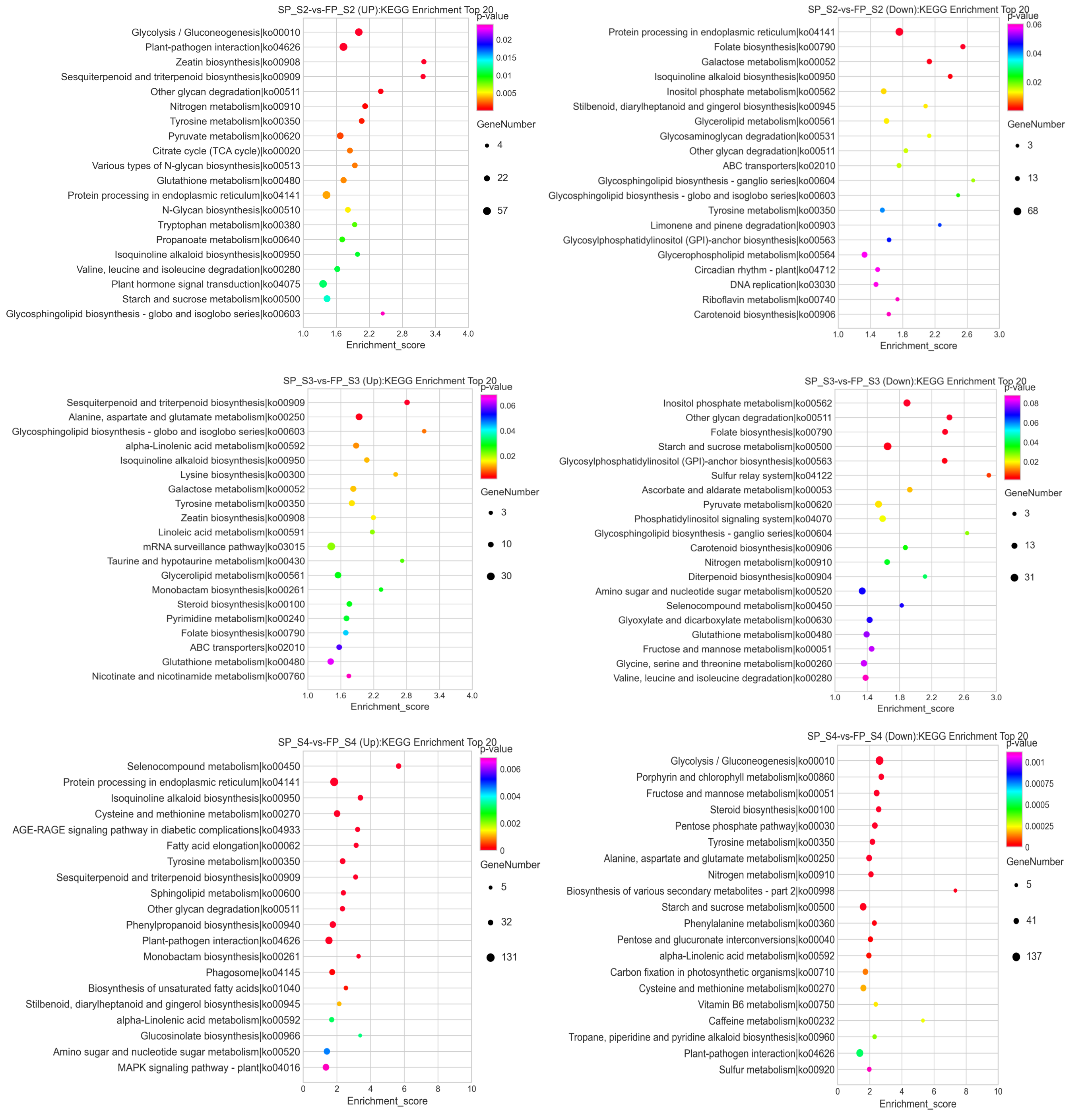
**

**Supplementary Figure 5. Scatter plot of enriched KEGG pathways (top 20) of up-and down-regulated DEGs at three periods of patchouli roots under continuous cropping stress.**

**
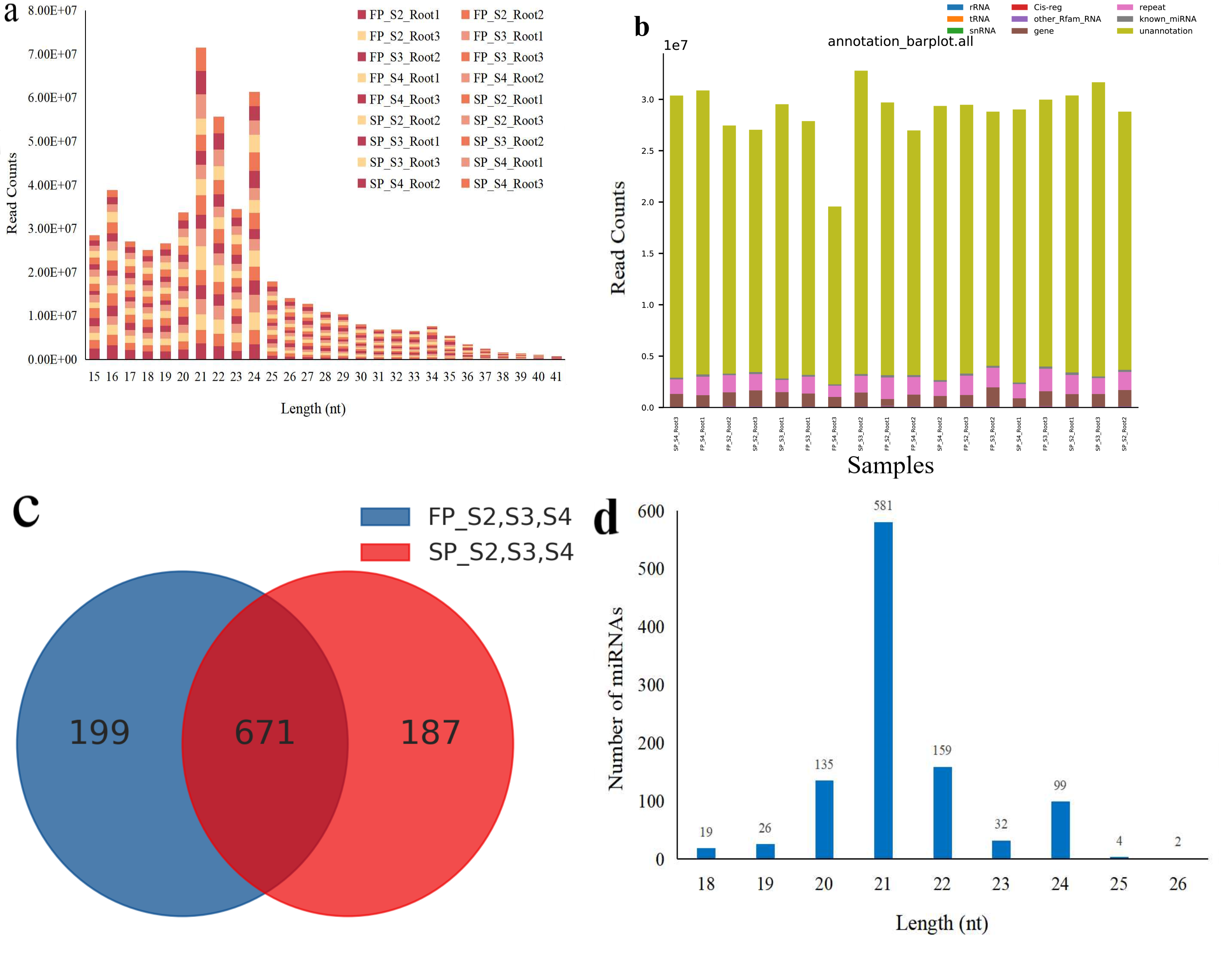
**

**Supplementary Figure 6. Overview of patchouli microRNAome.** (a) Length distribution of all reads from 18 samples. (b) Type classification of all sRNAs. The read counts of different RNA classes, including snRNA, tRNA, rRNA, Cis-reg, gene, repeat, other Rfam RNA, known miRNA and unannotation, were shown in histogram. (c) Venn diagrams of miRNAs expression overlap between SP and FP. (d) Length distribution of the identified miRNAs.


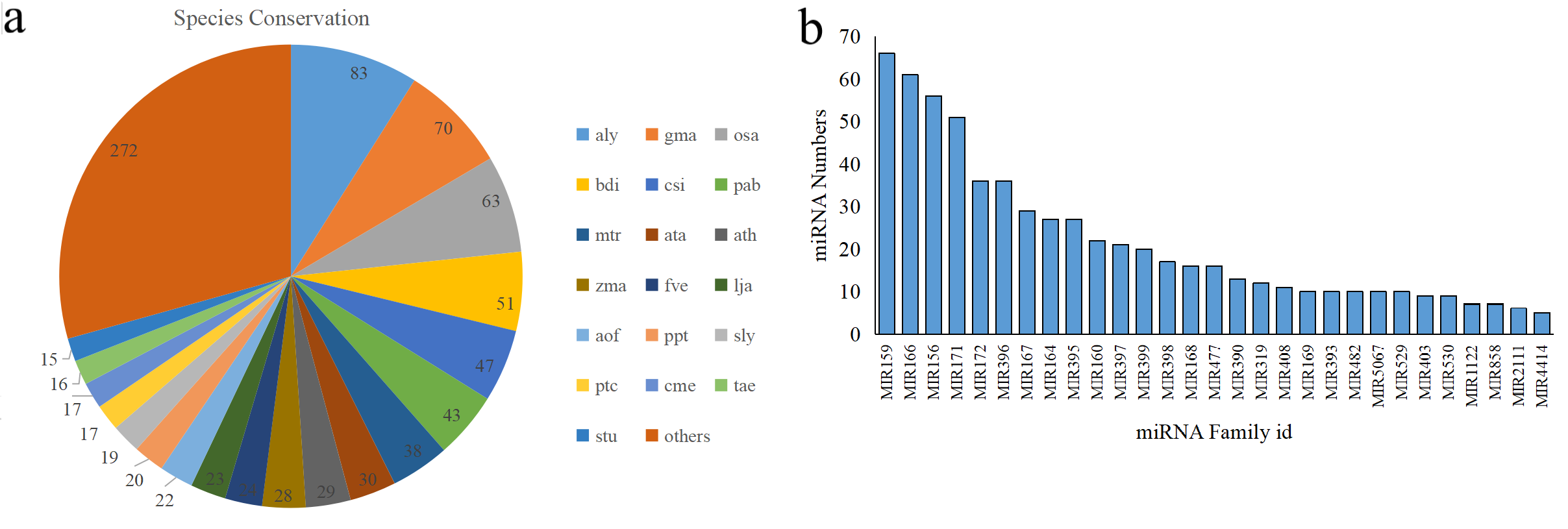


**Supplementary Figure 7. Classification and conservation analysis of miRNA families.** (a) Classification of miRNAs into classical families (miRNA families containing more than 5 miRNAs were listed). (b) Conservation analysis of all miRNAs. The species abbreviations: *Arabidopsis lyrata* (aly), *Glycine max* (gma), *Oryza sativa* (osa), *Brachypodium distachyon* (bdi), *Citrus sinensis* (csi), *Picea abies* (pab), *Medicago truncatula* (mtr), *Aegilops tauschii* (ata), *Arabidopsis thaliana* (ath), *Zea mays* (zma), *Fragaria vesca* (fve), *Lotus japonicus* (lja), *Asparagus officinalis* (aof), *Physcomitrella patens* (ppt), *Solanum lycopersicum* (sly), *Populus trichocarpa* (ptc), *Cucumis melo* (cme), *Triticum aestivum* (tae), *Solanum tuberosum* (stu).

**
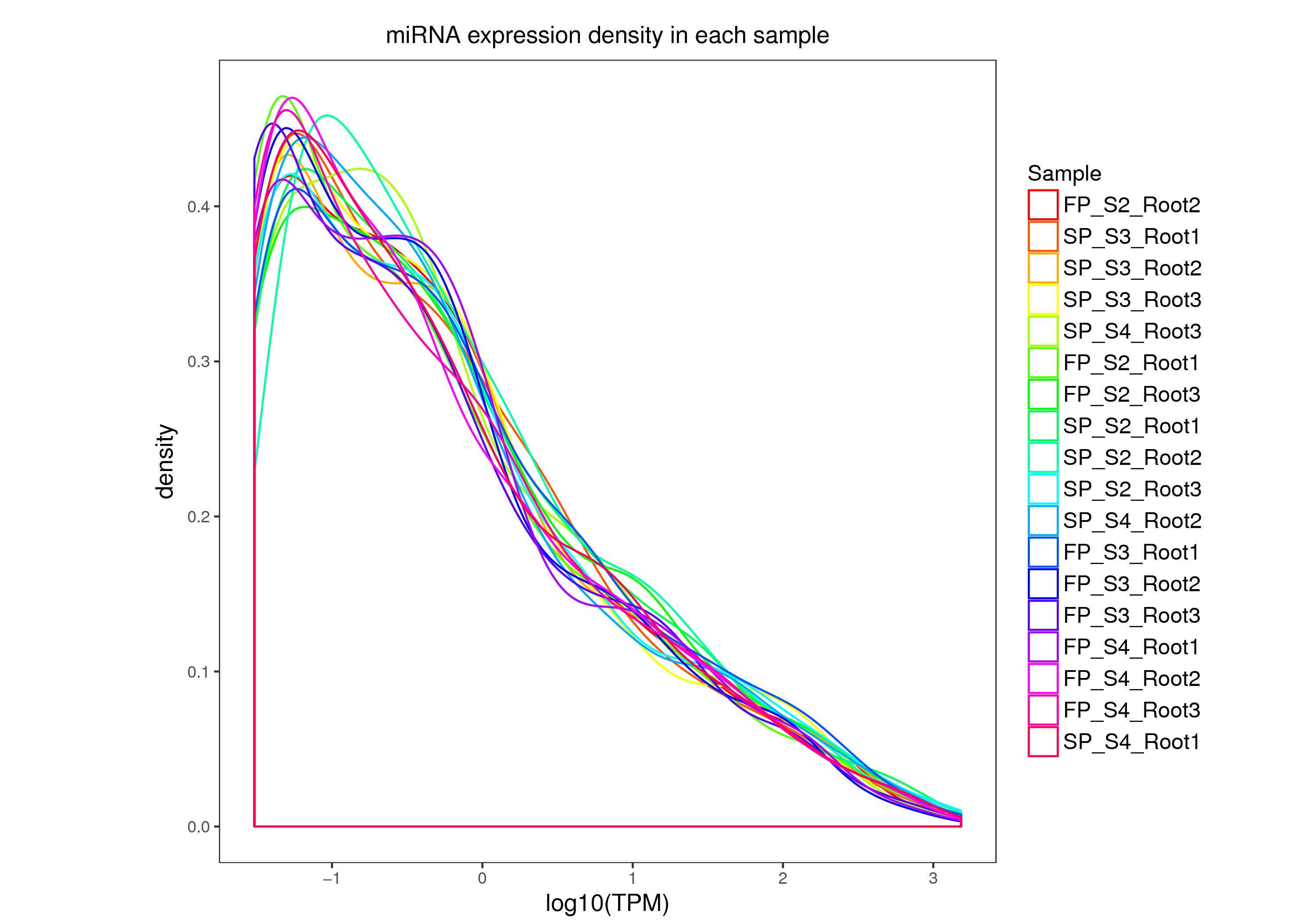
**

**Supplementary Figure 8. The transcripts per kilobase per million mapped reads density distribution of novel and conserved miRNAs in the 18 libraries.**

**
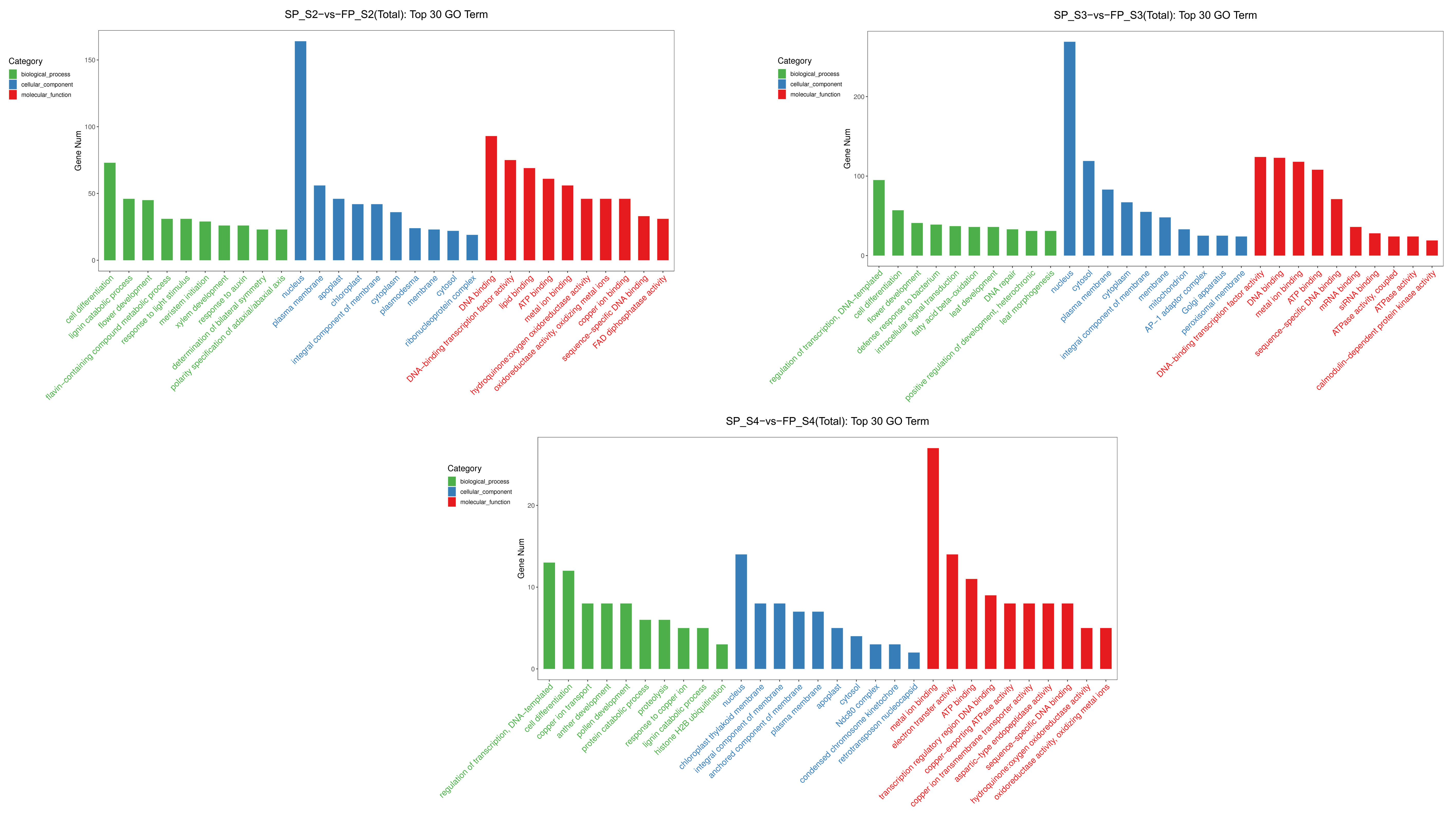
**

**Supplementary Figure 9. The 30 most significant terms in the GO enrichment analysis of the predicted target genes.**

**
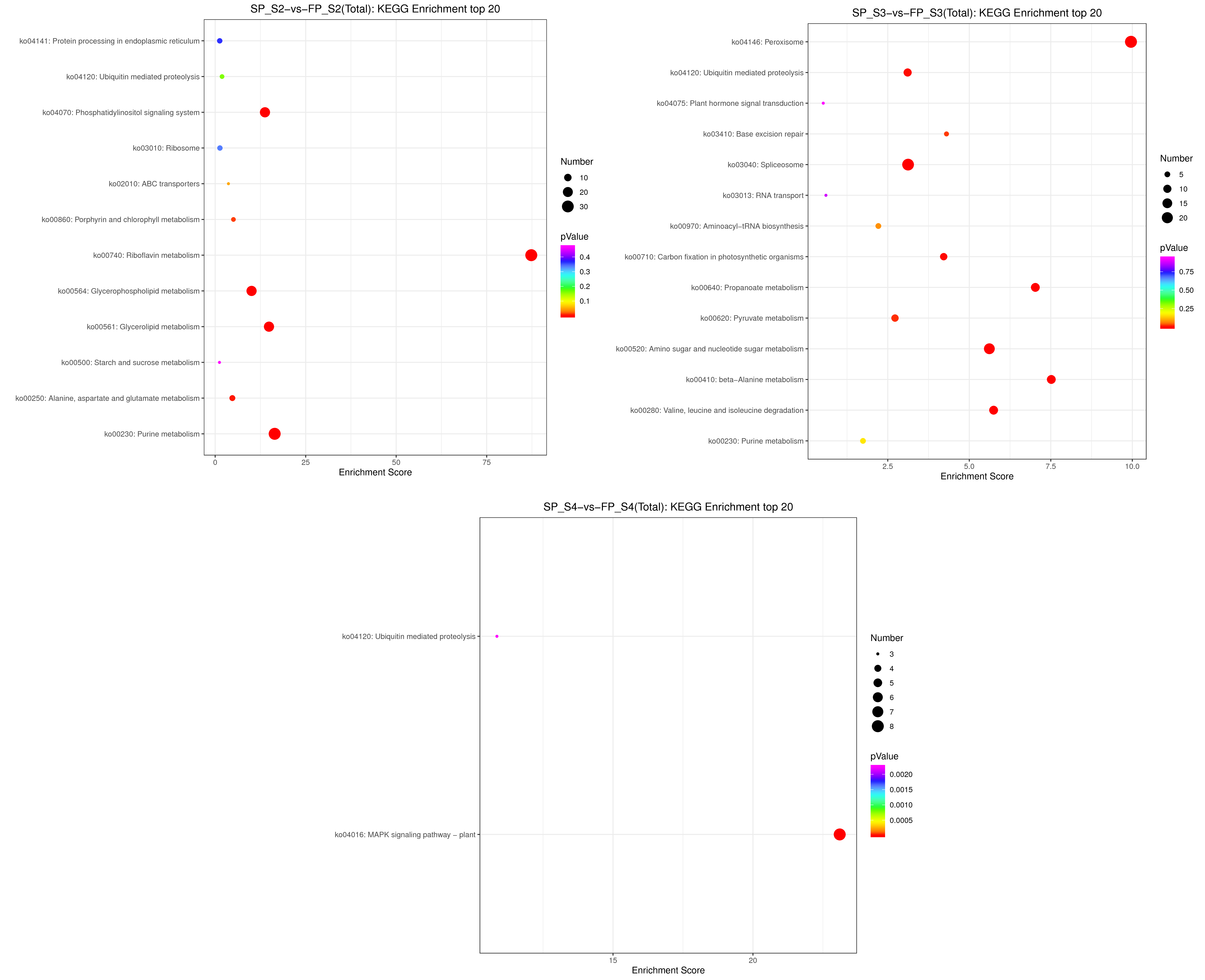
**

**Supplementary Figure 10. KEGG enrichment analysis for all predicted target genes.** The most significantly enriched KEGG pathways are shown. Circles indicated the gene number in each KEGG pathway. The color bar indicated the range of P value.
